# Long-Term Efficacy of Psychosocial Treatments for Adults With Attention-Deficit/Hyperactivity Disorder: A Meta-Analytic Review

**DOI:** 10.3389/fpsyg.2018.00638

**Published:** 2018-05-04

**Authors:** Carlos López-Pinar, Sonia Martínez-Sanchís, Enrique Carbonell-Vayá, Javier Fenollar-Cortés, Julio Sánchez-Meca

**Affiliations:** ^1^Department of Psychobiology, University of Valencia, Valencia, Spain; ^2^Department of Basic Psychology, University of Valencia, Valencia, Spain; ^3^Department of Psychology, University of Loyola, Andalucía, Spain; ^4^Department of Basic Psychology and Methodology, University of Murcia, Murcia, Spain

**Keywords:** meta-analysis, adult ADHD treatment, psychosocial treatment, long-term efficacy, cognitive-behavioral therapy, dialectical-behavior therapy, mindfulness-based cognitive therapy

## Abstract

**Background:** Recent evidence suggests that psychosocial treatments, particularly cognitive-behavioral therapy (CBT), are effective interventions for adult attention deficit hyperactivity disorder (ADHD). The objective of this review was to determine the long-term efficacy of psychosocial interventions in improving clinically relevant variables, including ADHD core symptoms, clinical global impression (CGI), and global functioning.

**Methods:** In total, nine randomized controlled trials and three uncontrolled single-group pretest-posttest studies were included. The data from these studies were combined using the inverse variance method. Heterogeneity and risk of bias were assessed. Subgroup analyses and meta-regressions were performed, to determine the influence of different potential moderator variables (risk of bias, medication status, follow-up length, therapy type and setting, and control group type) on effect size (ES) estimates.

**Results:** Up to 680 of a total of 1,073 participants assessed pre-treatment were retained at follow-up. Treatment groups showed greater improvement than control groups in self-reported total ADHD symptoms, inattention, and hyperactivity/impulsivity, in addition to CGI and global functioning. Blind assessors also reported a large ES in within-subject outcomes. Studies using dialectical behavioral therapy (DBT) in a group setting, with active control matching, and that were rated as having an unclear risk of bias, achieved significantly lower ES estimates for most outcomes. Treatment effectiveness, according to the CGI measure, and global functioning were significantly increased when the percentage of medicated participants was greater.

**Conclusions:** Our results indicate that the post-treatment gains reported in previous reviews are sustained for at least 12 months. Nevertheless, these results must be interpreted with caution, because of a high level of heterogeneity among studies and the risk of bias observed in the majority of outcomes. Thus, these findings indicate that psychological interventions are a highly valuable and stable clinical tool for the treatment of core symptoms and global functioning in adults with ADHD.

## Introduction

### Rationale

ADHD is a childhood-onset neurodevelopmental disorder characterized by developmentally inappropriate levels of inattention, hyperactivity, and impulsivity (American Psychiatric Association., 2013). The disorder affects 2.5–2.8% of the adult population (Simon et al., 2009; Fayyad et al., 2016), and is associated with significant impairment in academic (Kuriyan et al., 2013; Voigt et al., 2017), health (Nigg, 2013; Brevik et al., 2017), occupational (Kirino et al., 2015; Hechtman et al., 2016), and social (Das et al., 2012) domains. It is also related to the development of other comorbid conditions such as learning disorders (Knouse et al., 2012; Duda et al., 2015); oppositional defiant disorder (Reimherr et al., 2013); anxiety disorders (Cadman et al., 2016); substance use disorder (Capusan et al., 2016); and borderline personality disorder (Matthies and Philipsen, 2014), among others. In addition, the risk of suicide is significantly higher in adults with ADHD (Barbaresi et al., 2013), even after controlling for other comorbid disorders (Stickley et al., 2016). Thus, the need to treat ADHD is evidenced by the significant impact that the disorder has on the different areas of adult life. Pharmacotherapy is the first-line treatment for adults with ADHD with either moderate or severe level of impairment (National Institute for Health and Care Excellence, 2008). Stimulant drugs exhibit a moderate-to-large effect size (ES) (standardized mean difference [SMD] = 0.72) on ADHD symptoms (Castells et al., 2011; Epstein et al., 2014), while for non-stimulant drugs (e.g., atomoxetine), ES is low-to-moderate (SMD = 0.39) (Faraone and Glatt, 2010). Additionally, pharmacotherapy has long-term beneficial effects (Fredriksen and Peleikis, 2016). Nevertheless, drug therapy has significant limitations, since it is often associated with adverse effects and a high dropout rate (Cunill et al., 2016), and many individuals only exhibit partial responses (Wilens et al., 2002). Moreover, a combination of both psychosocial and medication treatment has proven more effective than drugs alone (Safren et al., 2005; Emilsson et al., 2011; Young et al., 2015), and is also associated with improved treatment adherence (Cunill et al., 2016).

Although they share therapeutic components, different psychosocial approaches have been designed or adapted for the treatment of adult ADHD, including: (1) Cognitive-behavioral therapy (CBT), which aims to develop behavioral strategies to compensate for core neuropsychiatric deficits and to change dysfunctional thinking styles (Safren et al., 2004); (2) Dialectical behavioral therapy (DBT), which is a CBT-based approach initially developed by Linehan (1993) for the treatment of borderline personality disorder, and was later modified to address the specific needs of adult ADHD. DBT aims to both promote the acceptance and validation of ADHD-related symptoms, and teach the skills required for change and self-management (Hesslinger et al., 2002); (3) Mindfulness meditation training, which is a type of meditative technique that emphasizes a compassionate and non-reactive attitude toward one's thoughts, emotions, and body state (Zylowska et al., 2008), and mindfulness-based cognitive therapy (MBCT), which is a combination of CBT and mindfulness; (4) Cognitive training (CT), which is premised on the notion that the key brain networks involved in ADHD can be strengthened, and the cognitive processes they subserve improved, through controlled exposure to information processing tasks (Vinogradov et al., 2012); and (5) Neurofeedback (NFB), which is a variant of EEG biofeedback that aims to promote self-regulation of specific brain activity patterns in an operant conditioning paradigm (Hammond, 2007).

Previously meta-analytic reviews have found that psychosocial interventions are effective at the end of the treatment, with moderate-to-large ES estimates on inattention and total ADHD symptoms and also on clinical global impression (CGI) and global functioning, which were reduced to small-to-moderate ES estimates for comparisons with active control groups, and only increased to large when within-subject data were analyzed, with small-to-moderate effects on hyperactivity/impulsivity symptoms (Linderkamp and Lauth, 2011; Cairncross and Miller, 2016; Jensen et al., 2016; Young et al., 2016; Knouse et al., 2017) (Table 1). Their results also varied depending on the source of information, since some reviews suggested that no significant effect was achieved (Jensen et al., 2016), while others found a moderate ES, according to blind evaluators (Knouse et al., 2017). In conclusion, psychosocial interventions have been found to improve ADHD symptoms and other clinically relevant variables in adults with ADHD at the end of treatment. However, none of these reviews examined whether the gains were maintained months after the end of treatment. In a disorder that tends to be chronic such as ADHD, the stability of improvements is one of the key features of an intervention, since the psychotherapy is aimed at the long-term modification of certain pathological behaviors and thoughts that cause nuclear symptoms to generate a greater impairment. Likewise, the efficacy of different therapy options (e.g., CBT, DBT, MBCT, etc.) have not been compared and several recently published significant studies have not been included in some of these reviews.

**Table 1:** Summary of characteristics of previous meta-analytic reviews.

**Reference**	**Years included**	**Studies included**	**Design**	**Treatment**	**Control**	**Main results**
Cairncross and Miller, 2016	Until 2014	3	RCT (*n* = 3)	MBCT (*n* = 3)	Waitlist (*n* = 3)	ADHD symptoms: SMD = −0.91 95% CI (−1.41 to −0.42); *I^2^* = 89.73
Jensen et al., 2016	Until 2014	2	RCT (*n* = 2)	CBT (*n* = 2)	TAU (*n* = 2)	ADHD symptoms (self-reported): SMD = −1.00 95% CI (−1.50 to −0.50) ADHD symptoms (clinician-reported): SMD = −0.60 95% CI (−1.30 to 0.10)
Knouse et al., 2017	Until 2015	32	RCT (*n* = 18) Uncontrolled pre-post studies (*n* = 12)	All considered CBT	Active (*n* = 5) Not active (*n* = 13)	ADHD symptoms (self-reported and assessor reported) from within-subject data: SMD = 1.00 95% CI (0.84 to 1.16); SMD = 1.40 95% CI (1.10 to 1.71) ADHD symptoms (self-reported) vs. control: SMD = 0.65 95% CI (0.44 to 0.86) CGI (assessor-reported) from within-subject data: SMD = 1.12 95% CI (0.79 to 1.43) Functioning (self-reported) from within-subject data and vs. control: SMD = 0.73; 95% CI (0.46 to 1.00); SMD = 0.51 95% CI (0.23 to 0.79)
Linderkamp and Lauth, 2011	Until 2010	12	RCT (*n* = 4) Uncontrolled pre-post studies (*n* = 8)	CBT (*n* = 8) DBT (*n* = 2) Psycho-education (n = 1)	Active (*n* = 1) Waitlist (*n* = 3)	All outcomes averaged: SMD = 0.84 95% CI (0.64 to 1.04)
Young et al., 2016	Until 2014	9	RCT (*n* = 8)	CBT (*n* = 8)	Active (*n* = 4) Waitlist (*n* = 5)	ADHD symptoms (CBT vs. Wait list): SMD = 0.76 95% CI (0.21 to 1.31); *I^2^* = 63% ADHD symptoms (CBT vs. Active control): SMD = 0.43 95% CI (0.14 to 0.71); *I^2^* = 31%

A number of studies have evaluated the efficacy of such interventions in the long-term, and demonstrated that it is maintained from 3 to 12 months after the end of the treatment (Safren et al., 2010; Salakari et al., 2010; Emilsson et al., 2011; Pettersson et al., 2014; Fleming et al., 2015; Salomone et al., 2015; Young et al., 2015; Cherkasova et al., 2016; Gu et al., 2017; Nasri et al., 2017), although the magnitudes of the reported effects are heterogeneous. The largest clinical trial so far published in the field found that groups receiving psychosocial therapy had superior outcomes to active control groups at follow-up only in the CGI measure, but not in ADHD symptoms (Philipsen et al., 2015). Other studies have also reported the maintenance of therapeutic achievements, according to the CGI measure (Safren et al., 2010; Young et al., 2015). Moreover, improvements in global functioning are also maintained, according to some studies (Emilsson et al., 2011; Fleming et al., 2015; Young et al., 2015; Morgensterns et al., 2016), but not others (Pettersson et al., 2014). To date, no meta-analytical review of the long-term efficacy of psychosocial treatment in adults with ADHD has been performed.

### Objectives

The main purpose of this review was to investigate if the observed post-treatment efficacy of psychological interventions on the nuclear symptoms of adults with ADHD was maintained from 3 months onwards after their termination. Similarly, the study aimed to ascertain if the post-treatment gains in CGI and global functioning were also sustained. Finally, we sought to explore how different variables (e.g., outcome measure source, within-study risk of bias, therapy type and setting, control group type, medication status, and follow-up length) moderate ES estimates for each outcome.

### Research questions

This study aims to answer the following research questions:

- Are therapeutic gains from the psychosocial treatments maintained at follow-up in adults with ADHD?
- To what extent do variables, such as therapeutic approach, medication status, or type of control group (among others), influence the maintenance of achievements?

## Methods

This review adheres to the Preferred Reporting Items for Systematic Reviews and Meta-analyses (PRISMA) guidelines (Liberati et al., 2009). A detailed checklist can be seen in the Supplementary Table 1.

### Study design

Randomized controlled trials (RCTs) and uncontrolled single-group pretest-posttest studies were included in the analysis. Although uncontrolled single-group pretest-posttest studies raise issues of internal validity, some authors in the meta-analytic area advocate their use in systematic reviews when there are few RCTs in a given field. For example, Hunter and Schmidt (2004) and Petticrew and Roberts (2005) supported the use of within-group designs in a meta-analysis when there are ethical or other reasons that hinder or prevent the use of control groups.

### Participants, interventions, comparators, and outcomes included

The following eligibility criteria were used in this review, detailed using the PICO approach (O'Connor et al., 2011).

#### Participants

Participants were required to meet the DSM-IV (4th ed., text. rev., American Psychiatric Association., 2000) or DSM-5 (5th ed., American Psychiatric Association., 2013) criteria for ADHD in adults. The studies should specify a detailed protocol of the diagnostic assessment. All participants had to be over 18 years old. Studies using a sample in which participants presented psychotic disorders, bipolar disorder, severe active addictions at the time of treatment, or clinically significant personality disorders (Axis II) were excluded; however, the existence of other Axis I disorders (mood disorders, anxiety disorders, etc.) was not a reason for exclusion.

#### Intervention

Studies in which at least one of the experimental groups received a psychosocial treatment specifically designed for ADHD, either supported by pharmacotherapy or not, were included. Such treatment had to be adequately described and detailed.

#### Comparisons

For the between-groups outcomes, studies in which at least one of the groups (control group) did not receive a specific psychotherapy for ADHD were included. The control group could be: (1) An active control group, in which participants received support from a therapist, in a group or individual non-directive sessions in which no specific strategy was discussed, or even non-specific interventions for ADHD such as relaxation training; (2) A treatment as usual (TAU) group, in which all participants received the usual treatment, which included both pharmacological and potentially also some non-pharmacological treatments; (3) A waiting-list group, in which participants were waiting without receiving any psychosocial treatment, although some, but not all, participants received pharmacotherapy.

#### Outcomes

Studies were required to perform a follow-up assessment at least 3 months after the end of the treatment, which had to include an ADHD symptoms severity scale, since this was used as the primary outcome measure for our review. CGI and global functioning were secondary outcomes. CGI (National Institute of Mental Health, 1985) is a three-item observer-rated scale that measures illness severity, global improvement or change, and therapeutic response. It has been proven as a robust measure of efficacy in clinical trials; therefore, it has been widely used in many treatment evaluation studies. The global functioning outcome provides a measure of the impact that symptoms have on daily functioning in the vital domains (e.g., social, familial, work, personal, and academic, among others). Instruments, such as the Sheenan Disability Scale (Sheenan et al., 1996) or the RATE-S (Young and Ross, 2007) were included in that outcome category.

### Systematic review protocol

In accomplishing this systematic review, no previous protocol was carried out.

### Search strategy

A systematic literature search was performed in MEDLINE (via PubMed) and Scopus. No date limit was established. The last search was conducted on September 4, 2017. The search terms used are detailed in Supplementary Table 2. In addition, reference lists of retrieved relevant articles were screened.

### Data sources, study selection, and data extraction

**Figure 1:**
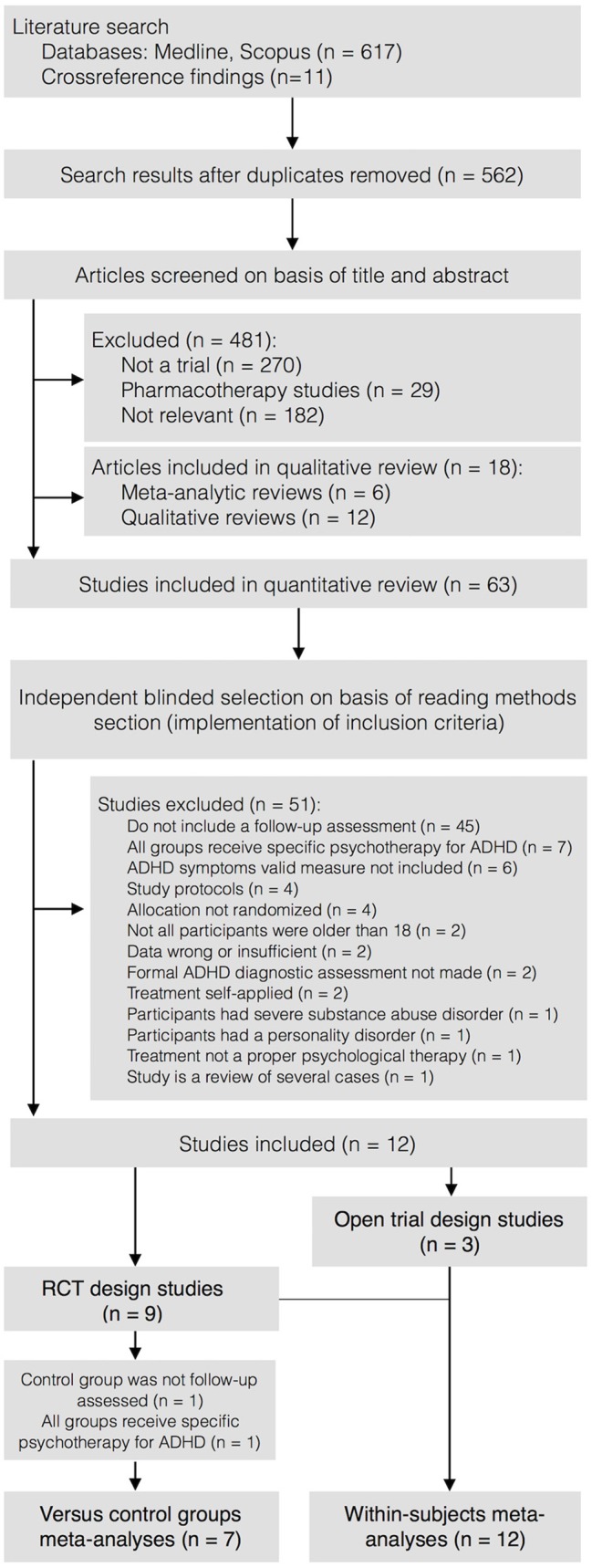
Selection process diagram.

The selection process is illustrated in Figure 1. After removing duplicates, the first author conducted a screening search of relevant articles, based on reading the title and abstract. Then, the two first authors independently applied the eligibility criteria to the methods section of the articles, which were previously masked by a third researcher. Agreement between the two researchers was acceptable (κ = 0.81). Inconsistencies between the assessors were solved by consensus. The final selection of the studies was based on reading the full-text articles.

Data were extracted and coded in a spreadsheet so that all study characteristic items described in the Cochrane Handbook were covered (Higgins and Green, 2011). This sheet was pilot-tested with five studies, to improve its fit to the sample characteristics. Data extraction and coding were performed independently by the two first authors. The codebook can be requested from the corresponding author. Kappa coefficients of inter-rater agreement were excellent for qualitative moderator variables (average κ = 0.88; range = 0.75–0.93), as well as for continuous moderator variables (average intra-class correlation *r* = 0.95; range = 0.91–1.0). Disagreements were resolved by consensus. Four authors were contacted to request additional information. All of them responded, and data were provided by three of them. To reduce bias, data in included studies were searched for duplicates.

The following data were extracted from each study (Table 2): (1) methodological characteristics: study design, sample size at every assessment point, and follow-up length; (2) participant characteristics: percentage of participants receiving ADHD medication; (3) intervention characteristics: type of therapy, therapy setting, and number of sessions; (4) comparison group characteristics: control group type; (5) outcomes: total ADHD symptoms, inattention and hyperactivity/impulsivity symptoms, CGI, and global functioning; and (6) outcome characteristics: measure source (self-rated or blind assessor-rated) for all outcomes.

**Table 2:** Summary of characteristics of included studies.

	**Participants**			**Intervention**			**Control**	**Outcomes**		
	**Pre-treatment (n)**	**Follow-up (n)**	**% on ADHD meds**	**Therapy**	**Setting**	**Sessions**		**ADHD measure source**	**Follow-up length (months)**	**Other outcomes considered**
Cherkasova et al., 2016	46	34	0	CBT	Combined	12	–	Self-report	6	Global functioning
	42	26	100							
Emilsson et al., 2011	27/27	8/13	100	CBT	Combined	15	TAU	Blind assessor/Self-report	3	CGI; Global functioning
Fleming et al., 2015	17/16	16/16	70.61/18.83	DBT	Combined	8	Waitlist	Self-report	3	Global functioning
Gu et al., 2017	30/26	28/26	71.42/72.91	MBCT	Individual	6	Waitlist	Self-report	3	–
Morgensterns et al., 2016	98	58	74.73	DBT	Group	14	–	Self-report	3	–
Nasri et al., 2017	18	18	72	CBT+DBT	Group	14	–	Self-report	6	–
Pettersson et al., 2014	14/18	11/0	42.92/50	CBT–G	Group	10	Waitlist	Self-report	6	Global functioning
Philipsen et al., 2015	107/110	69/70	100	DBT	Group	12	Active	Blind assessor/Self-report	12	CGI
	109/107	59/45	0							
Safren et al., 2010	43/43	38/32	100	CBT	Individual	12	Active	Blind assessor/Self-report	12	CGI
Salakari et al., 2010	29	25	66	CBT	Group	10-11	–	Self-report	6	–
Salomone et al., 2015	24/27	15/14	33.33/22.20	BFB	Individual	N/E	Active	Self-report	3	–
Young et al., 2015	48/47	27/32	100	CBT	Combined	15	TAU	Blind assessor/Self-report	3	CGI; Global functioning

### Data analysis

For data analysis, Review Manager software (version 5.3) from the Cochrane Collaboration and Comprehensive Meta Analysis (version 3.3.070) were used. Long-term reduction in the severity of inattention, hyperactivity/impulsivity, and total ADHD symptoms were considered the primary outcomes, while CGI and global functioning were secondary outcomes. For the between-group (psychosocial treatments vs. control groups) outcomes, the effect size index was defined as the difference between the average pretest-follow-up change of the experimental and control groups, divided by a pooled estimate of the pretest standard deviations of the two groups. In addition, a correction factor for small samples sizes was also applied (Morris, 2008; see Supplementary Figure 1). For within-subject (pretest to follow-up) single-group studies, the effect size was defined as the average pretest-follow-up change, divided by the pretest standard deviation, and with a correction factor for small sample sizes (Morris, 2000; see Supplementary Figure 2). For estimating the variances of both effect size indices, the Pearson correlation coefficient between the pretest and follow-up measures must be available. As this figure was not reported in the studies, a value of 0.70 was assumed for *r*, as recommended by Rosenthal (1991). Following the rule of thumb suggested by Cohen (1988), ES values of 0.20, 0.50, and 0.80 were considered small, moderate, and large, respectively.

The results of individual studies, weighted by their inverse variance, were combined for each outcome. A random-effects model was chosen because of a suspicion of a high heterogeneity between the studies. A 95% confidence interval (CI) was calculated for each outcome. The consistency of effect sizes was assessed using the *I*^2^ index (Higgins et al., 2003), which describes the percentage of total variation across studies that is due to heterogeneity, rather than chance. *I*^2^ values of 25, 50, and 75% can be interpreted as reflecting low, moderate, and high heterogeneity, respectively (Higgins et al., 2003).

To determine the internal validity of each study, the risk of bias was assessed by the first author, covering the items described in The Cochrane Collaboration's tool for assessing risk of bias (Higgins et al., 2011): (a) the adequacy of randomization and concealment of allocation (selection bias), where a comparison group was available; (b) the blinding of the outcome assessors (blinding of the therapists could not be assessed in studies that evaluated psychosocial treatments); (c) the incomplete outcome data (attrition bias); (d) the selective reporting of the outcomes (reporting bias); and (e) the medication stabilization (other sources of bias). This assessment was supervised by the second author and discrepancies were resolved by consensus. Studies were not excluded based on the result of the evaluation of the risk of bias, but they were divided into subgroups (high, unclear, or low risk of bias) and sensitivity analyses were performed to determine the influence of this variable on ES estimates.

Publication bias was assessed by visually examining the asymmetry in the funnel plots of each outcome and conducting the trim-and-fill method (Duval and Tweedie, 2000). This test trims the asymmetric studies from the right-hand side to locate the unbiased effect (in an iterative procedure), and then fills the plot by re-inserting the trimmed studies on the right, as well as including their imputed counterparts to the left of the mean effect. In addition, the Egger test (Egger et al., 1997) for testing the asymmetry of funnel plots was applied. This test assesses bias using the precision of each ES (the inverse of the standard error) to predict the standardized effect (ES divided by the standard error). Finally, the Fail-safe N (Rosenthal, 1979) was also calculated, which is the number of additional “negative” studies (with a null effect) that would be needed to increase the *P*-value for the meta-analysis to above 0.05.

Sub-group analyses (chi-square tests) were performed for each outcome, to assess the impact of the following categorical variables on ES estimates: (i) risk of bias in individual studies; (ii) therapy type; (iii) therapy setting; (iv) outcome measure source; (v) and control group type. Additionally, a meta-regression was carried out for each outcome with continuous variables, such as the percentage of participants in the experimental group under pharmacological treatment and the follow-up length, to ascertain the extent to which they predicted the ES.

## Results

### Study selection

From 236 records, nine RCTs and three uncontrolled single-group pretest-posttest studies were identified and included in the quantitative review, based on the reading of full-text reports (Figure 1). Two RCTs were excluded from between-groups, but included in within-subject meta-analyses, because the control group was not assessed at follow-up (Pettersson et al., 2014) and all groups received specific ADHD psychotherapy (Cherkasova et al., 2016). Thus, finally, seven and 12 studies were included in the between-groups and within-subject meta-analyses, respectively. The characteristics of the included studies are presented in Table 2, and a list of excluded studies and the reasons for exclusion are detailed in Supplementary Table 3. For the study by Philipsen et al. (2015), each treatment group was compared with the equivalent control group, based on medication status.

#### Participants

Up to 680 of 1,073 participants assessed pre-treatment were retained at follow-up. On average, 50.72% of participants were male, age was 34.41 years, and 63.70% of participants were taking medication for ADHD during the treatment.

#### Intervention

Half of the treatment groups underwent CBT (50%) and 25% DBT; while MBCT, Biofeedback (BFB), or a combination of CBT and DBT were applied for 8.33%. On average, 11.42 sessions were conducted. Group and individual treatments were delivered in 41.66 and 25% of studies, respectively, while both types of treatment were combined in 33.33%.

#### Comparison

An active control group was used in 42.92% of the studies, while 28.64% compared the intervention to a TAU or waitlist group.

#### Outcomes

Blinded assessors of the primary outcome measure were used in 33.33% of studies, while the remainder used only self-reported measures.

### Synthesized findings

#### All treatment vs. all control groups

**Figure 2:**
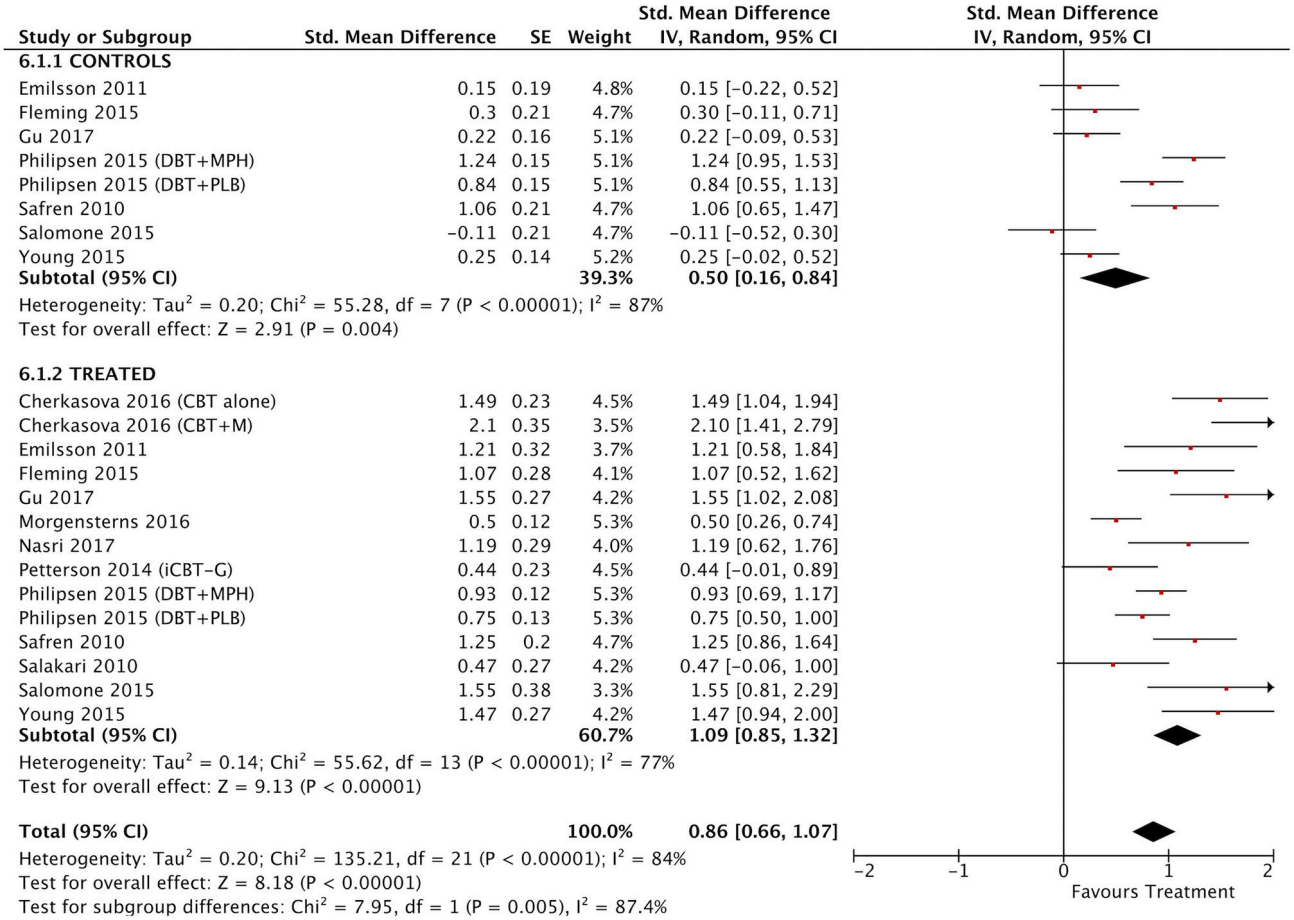
Forest plot for all treatment and control groups on self-reported total ADHD symptoms outcome.

**Figure 3:**
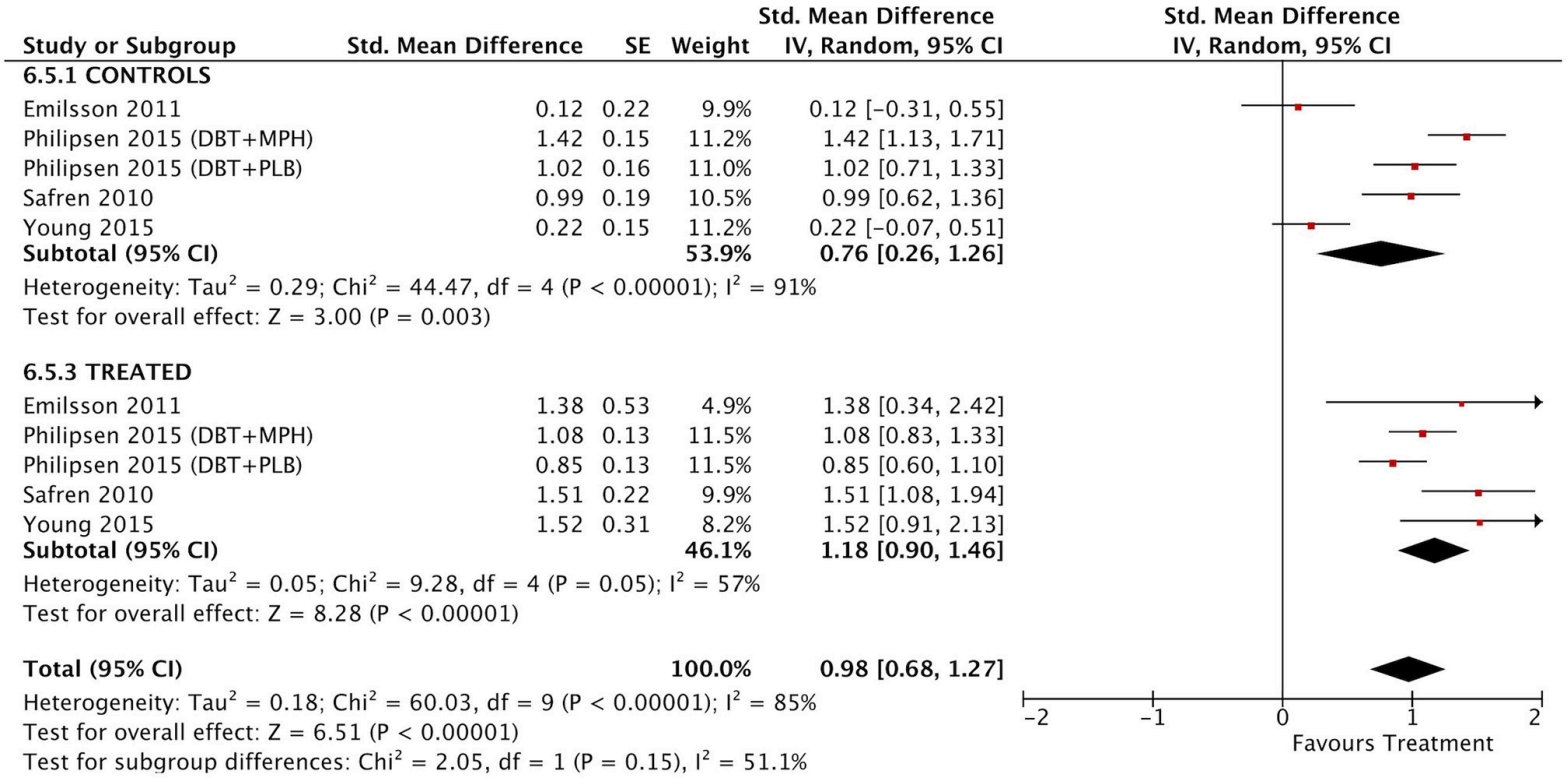
Forest plot for all treatment and control groups on blind assessor-reported total ADHD symptoms outcome.

Combining all treatment groups, significant differences were found between the subjects that received a psychosocial intervention and those in the combined control groups in self-reported total ADHD symptoms [$\chi_{\left(1\right)}^{2}$ = 7.95, *p* < 0.01], inattention [$\chi_{\left(1\right)}^{2}$ = 6.34, *p* = 0.01], and hyperactivity/impulsivity [$\chi_{\left(1\right)}^{2}$ = 10.90, *p* < 0.01], as well as in CGI [$\chi_{\left(1\right)}^{2}$ = 5.66, *p* = 0.02], and global functioning [$\chi_{\left(1\right)}^{2}$ = 5.60, *p* = 0.02] outcomes, in favor of the treated groups (Figure 2). Although the treated groups obtained a higher ES, differences in blind assessor rated total ADHD symptoms [$\chi_{\left(1\right)}^{2}$ = 2.05, *p* = 0.15], inattention [$\chi_{\left(1\right)}^{2}$ = 0.01, *p* = 0.91], and hyperactivity/impulsivity [$\chi_{\left(1\right)}^{2}$ = 2.37, *p* = 0.12] outcomes were not significant (Figure 3).

#### Between-group outcomes

**Figure 4:**
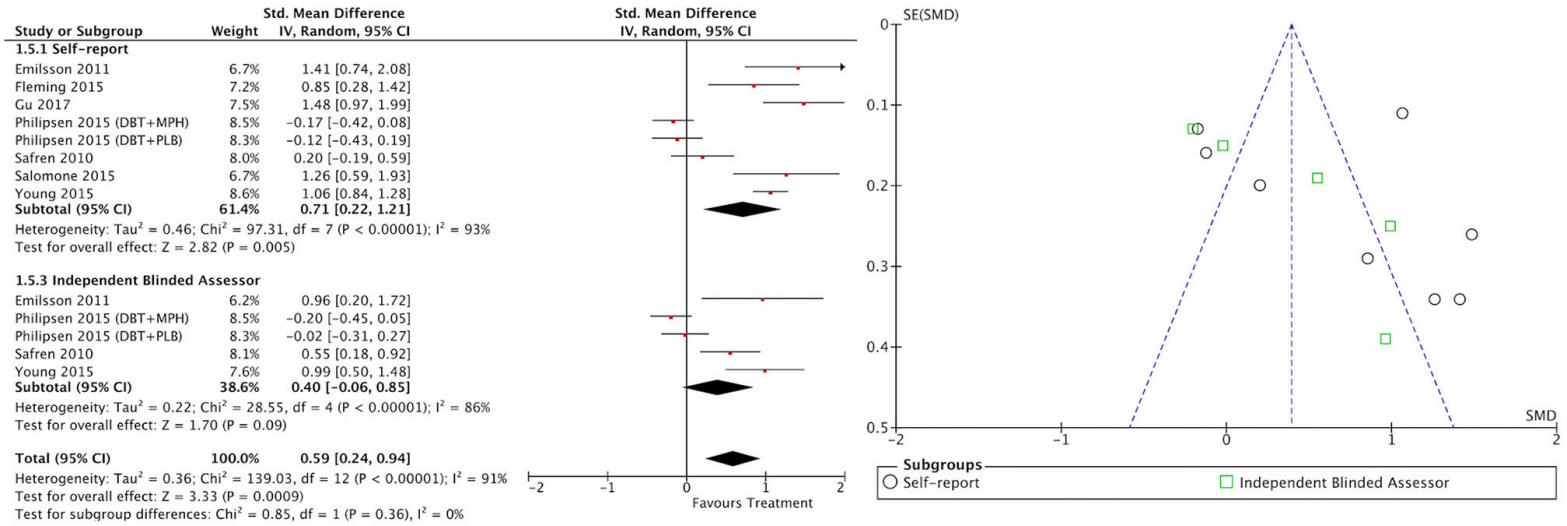
Forest and funnel plots for between-groups total ADHD symptoms outcome.

**Figure 5:**
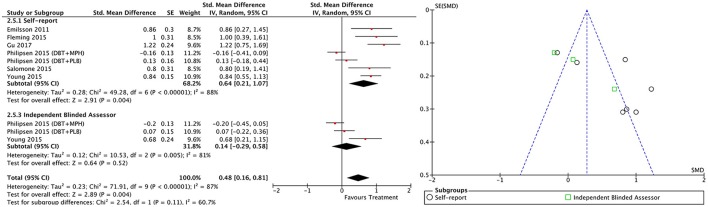
Forest and funnel plots for between-groups inattention symptoms outcome.

**Figure 6:**
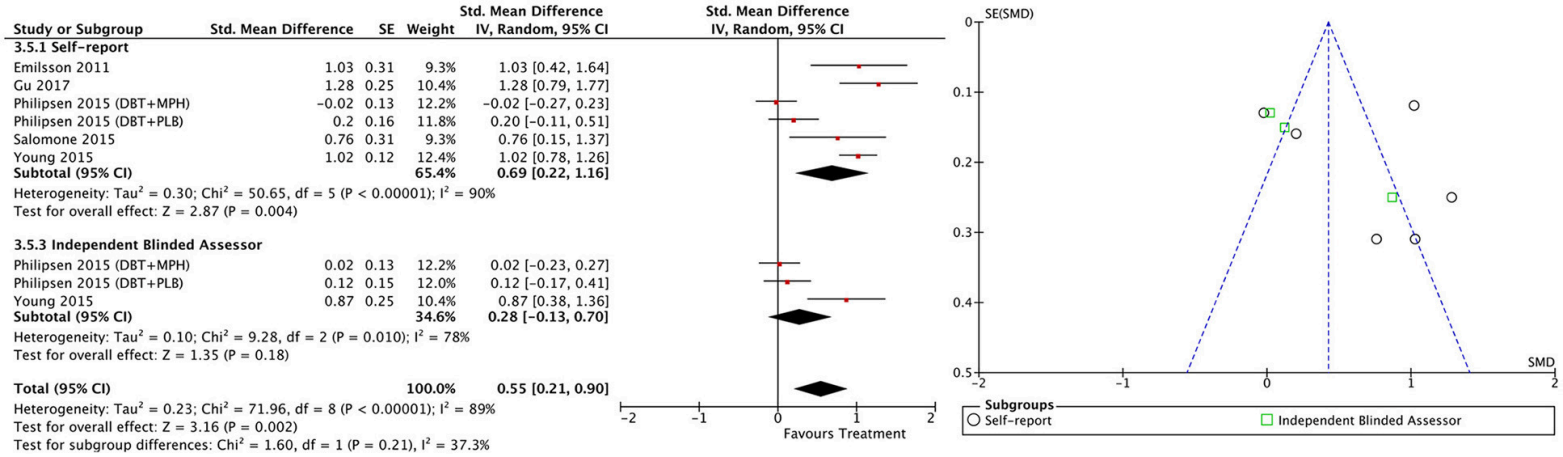
Forest and funnel plots for between-groups hyperactivity/impulsivity symptoms outcome.

**Figure 7:**
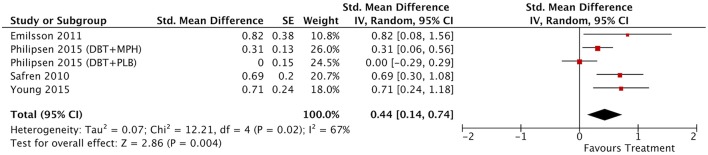
Forest plot for between-groups CGI outcome.

**Figure 8:**
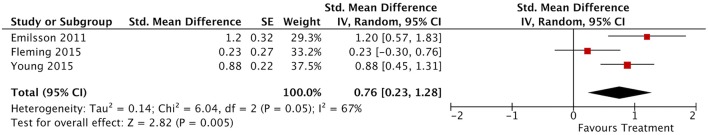
Forest plot for between-groups global functioning outcome.

Taking into account only data from the RCTs, treatment groups showed greater improvement than control groups in self-reported total ADHD symptoms (SMD = 0.71; 95% CI [0.22–1.21]), inattention (SMD = 0.64; 95% CI [0.23–1.01]), and hyperactivity/impulsivity (SMD = 0.66; 95% CI [0.18–1.14]) outcomes, for which ES estimates were medium-to-large (Table 3; Figures 4–6). In contrast, blind assessors reported small-to-moderate ES on total ADHD symptoms (SMD = 0.40; 95% CI [−0.06 to 0.85]), and hyperactivity/impulsivity (SMD = 0.28; 95% CI [−0.13 to 0.70]), and a small ES on inattention (SMD = 0.14; 95% CI [−0.29 to 0.58]) outcomes, but with confidence intervals including zero. Treatment efficacy measured by CGI was small-to-moderate (SMD = 0.44; 95% [0.14–0.74]) (Figure 7). Finally, a moderate-to-large ES (SMD = 0.76; 95% [0.23–1.28]) was achieved for self-reported global functioning (Figure 8). High heterogeneity was observed for all outcomes (Table 3).

**Table 3:** Standardized mean differences (SMD), 95% confidence intervals, heterogeneity analyses, and risk of bias for between-groups and within-subject outcomes.

		**Between-groups outcomes**						**Within-subject outcomes**					
**Outcome**	**Rater**	**Studies**	**N**	**SMD**	**95% CI**	**Hetero-geneity (*I^2^*)**	**Risk of bias**	**Studies**	**N**	**SMD**	**95% CI**	**Hetero-geneity (*I^2^*)**	**Risk of bias**
Total ADHD symptoms	Self-rated	8	513	0.71	0.22 to 1.21	93%	High	14	409	1.09	0.85 to 1.32	77%	High
	Blind assessor	5	382	0.40	−0.06 to 0.85	86%	Unclear	5	197	1.18	0.90 to 1.46	57%	Unclear
Inattention symptoms	Self-rated	7	446	0.64	0.21 to 1.07	88%	High	8	243	1.20	0.96 to 1.44	53%	High
	Blind assessor	3	282	0.14	−0.29 to 0.58	87%	Unclear	3	153	0.91	0.74 to 1.07	0%	Unclear
Hyperactivity/impulsivity symptoms	Self-rated	6	406	0.69	0.22 to 1.16	90%	High	7	227	0.83	0.59 to 1.08	64%	High
	Blind assessor	3	291	0.28	−0.13 to 0.70	78%	Unclear	3	149	0.67	0.49 to 0.85	24%	Unclear
Clinical Global Impression	Blind assessor	5	392	0.44	0.14 to 0.74	67%	Unclear	5	194	1.20	0.93 to 1.48	57%	Unclear
Global functioning	Self-rated	3	120	0.76	0.23 to 1.28	67%	High	4	102	0.58	0.25 to 0.92	72%	High

#### Within-subject outcomes

**Figure 9:**
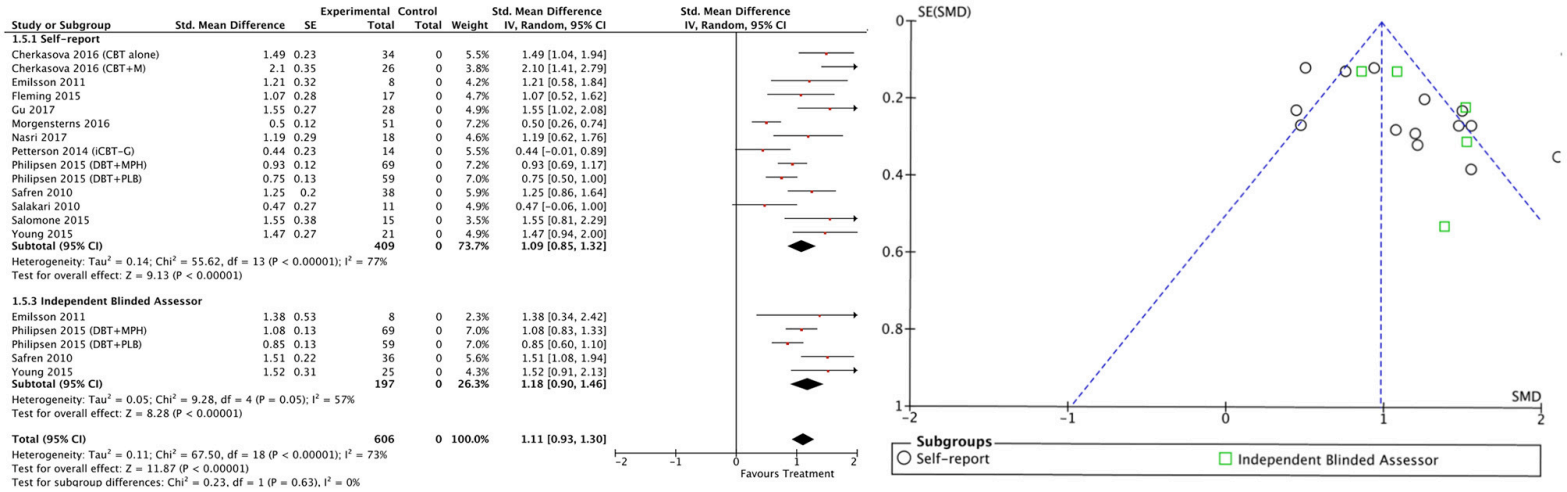
Forest and funnel plots for within-subject total ADHD symptoms outcome.

**Figure 10:**
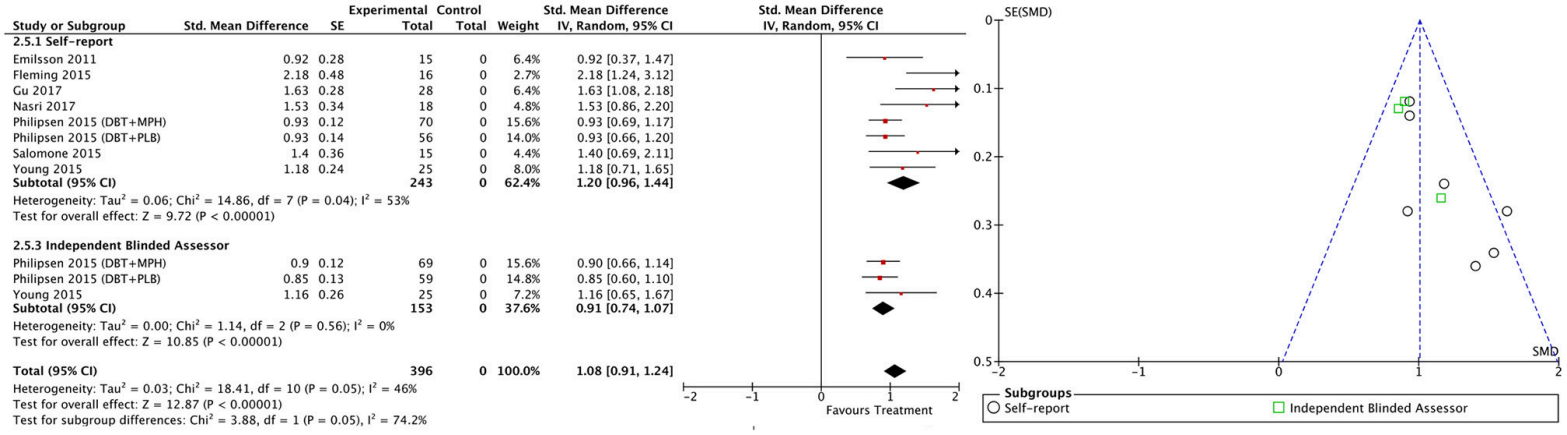
Forest and funnel plots for within-subject inattention symptoms outcome.

**Figure 11:**
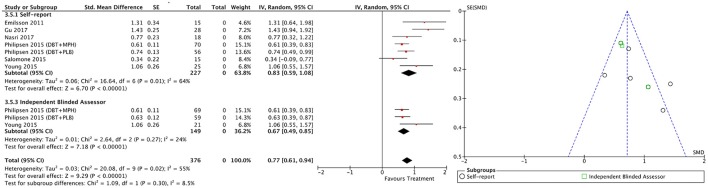
Forest and funnel plots for within-subject hyperactivity/impulsivity symptoms outcome.

**Figure 12:**
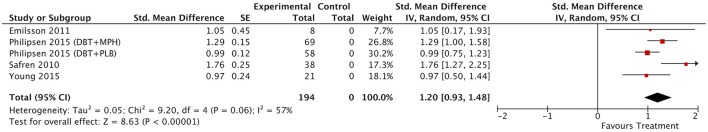
Forest plot for within-subject CGI outcome.

**Figure 13:**
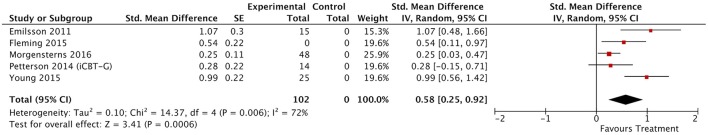
Forest plot for within-subject global functioning outcome.

The within-subject treatment ES estimates on all ADHD symptom outcomes were large, both for self-reported (SMD = 1.09; 95% CI [0.85–1.32] for total ADHD symptoms) and blind assessed (SMD = 1.18; 95% CI [0.90–1.46] for total ADHD symptoms) measures, except for hyperactivity/impulsivity symptoms as reported by blind assessors (SMD = 0.67; 95% CI [0.49–0.85]) (Table 3, Figures 9–11). The ES on CGI outcome was also large (SMD = 1.20; 95% CI [0.93–1.48]) (Figure 12), while that on global functioning was moderate-to-large (SMD = 0.58; 95% CI [0.25–0.92]) (Figure 13). *I*^2^ indices indicated greater homogeneity than for the between-groups outcomes (Table 3). Heterogeneity was zero and low for the blind assessor-rated inattention and hyperactivity/impulsivity outcomes, respectively, and high for self-rated total ADHD symptoms and global functioning, whereas it was moderate for the remaining outcomes.

### Risk of bias

All self-rated outcomes were rated with a high risk of bias, while the risk of bias summary for the blind assessor-rated outcomes was classified as unclear (Supplementary Tables 4–8). Only one study was assessed to have a low risk of bias for all outcomes, except for global functioning.

Publication bias indicators were identified for the between-groups outcomes. The results of the Egger's test were significant or marginally significant for total ADHD symptoms, inattention, and hyperactivity/impulsivity outcomes (Supplementary Table 9). Use of the Trim and Fill method decreased the confidence interval to below zero after trimming some studies in the self-reported and blind assessor-rated total ADHD symptoms and self-reported inattention outcomes. In contrast, between-groups CGI and global functioning outcomes are likely robust to publication bias. Similarly, fail-safe N results for self-reported total ADHD symptoms, inattention, and hyperactivity/impulsivity outcomes indicated that a number of studies between six and eight times higher than those included would be necessary for the estimated effect to be null.

Regarding within-subject outcomes, although the results of the Egger's test were significant for blind assessor-rated total ADHD symptoms, inattention, and hyperactivity/impulsivity outcomes, the confidence interval of the Trim and Fill method adjusted estimates remained above zero (Supplementary Table 10). In addition, the fail-safe N for those outcomes ranged from 42 to 157. No indicators of publication bias were apparent for self-reported total ADHD symptoms, inattention, or hyperactivity/impulsivity, or for CGI or global functioning outcomes.

### Moderator analyses

#### Risk of bias

Significant differences were found for between-groups total ADHD symptoms [$\chi_{\left(2\right)}^{2}$ = 11.74, *p* < 0.01], inattention [$\chi_{\left(2\right)}^{2}$ = 31.22, *p* < 0.01], and hyperactivity/impulsivity [$\chi_{\left(2\right)}^{2}$ = 31.40, *p* = 0.01] (Supplementary Table 11), as well as for within-subject inattention [$\chi_{\left(2\right)}^{2}$ = 8.23, *p* = 0.02] (Supplementary Table 12). ES estimates were significantly lower for studies rated as having an unclear risk of bias for all of these outcomes.

#### Therapy

DBT studies achieved significantly, or marginally significantly, lower ES estimates on between-groups total ADHD symptoms [_χ2(3)_ = 16.47, *p* < 0.01], inattention [_χ2(3)_ = 8, *p* = 0.05], and hyperactivity/impulsivity [_χ2(3)_ = 32.96, *p* < 0.01] (Supplementary Table 11), on within-subject total ADHD symptoms [_χ2(3)_ = 7.42, *p* = 0.06] (see Supplementary Table 12), and on between-groups CGI [_χ2(1)_ = 6.87, *p* < 0.01] and global functioning [_χ2(1)_ = 5.36, *p* = 0.02]. Biofeedback studies generated significantly lower ES estimates on within-subject hyperactivity/impulsivity symptoms [_χ2(3)_ = 17.10, *p* < 0.01].

#### Treatment setting

ES estimates were significantly, or marginally significantly, lower in studies using a group treatment setting on between-groups and within-subject total ADHD symptoms [_χ2(2)_ = 36.68, *p* < 0.01 and _χ2(2)_ = 15.83, *p* < 0.01, respectively], inattention [_χ2(2)_ = 29.21, *p* < 0.01 and _χ2(2)_ = 5.83, *p* = 0.05, respectively], and hyperactivity/impulsivity [_χ2(2)_ = 24.83, *p* < 0.01 and _χ2(2)_ = 5.75, *p* = 0.06, respectively] (Supplementary Tables 11, 12), as well as on between-groups CGI [_χ2(2)_ = 6.90, *p* = 0.03] and within-subject global functioning [_χ2(1)_ = 8.92, *p* < 0.01] outcomes, while the ES on the within-subject CGI outcome was significantly higher than that for the individual setting [_χ2(2)_ = 6.25, *p* = 0.04].

#### Measure source

A significantly lower ES on the within-subject inattention outcome was found for blind assessor measurement [_χ2(1)_ = 3.88, *p* = 0.05], while no significant differences were found for the remaining outcomes (Supplementary Tables 11, 12).

#### Control group

Active control-matched studies generated a significantly, or marginally significantly, lower ES on total ADHD symptoms [_χ2(2)_ = 5.71, *p* = 0.06], inattention [_χ2(2)_ = 11.61, *p* < 0.01], and hyperactivity/impulsivity [_χ2(2)_ = 16.46, *p* < 0.01], as well as for global functioning [_χ2(1)_ = 5.36, *p* = 0.02] (Supplementary Table 11).

#### Follow-up length

None of the meta-regressions were significant with respect to this variable, with a confidence level of 95% (Supplementary Tables 13, 14).

#### Percentage of participants on medication

A higher percentage of participants on medication predicted higher ES on between-groups and within-subject CGI (Coefficient = 0.01, 95% CI [0.00–0.01], *p* = 0.02; Coefficient = 0.01, 95% CI [0.00–0.01], *p* = 0.01) and on within-subject global functioning and total ADHD symptoms (Coefficient = 0.02, 95% CI [0.00–0.04], *p* = 0.01; Coefficient = 0.01, 95% CI [0.00 to 0.001]), while no significant meta-regressions were found for the remaining outcomes (Supplementary Tables 13, 14).

## Discussion

### Summary of main findings

Psychosocial treatments have exhibited post-treatment efficacy for both core and other symptoms in adults with ADHD (Cairncross and Miller, 2016; Jensen et al., 2016; Young et al., 2016; Knouse et al., 2017); however, no previous studies have systematically explored whether therapeutic improvements are maintained at follow-up assessment. In this study, a meta-analytic review was conducted to determine if treatment gains were sustained between 3 and 12 months after the end of treatment, for both core ADHD symptoms and other clinically relevant measures, and to what extent different moderator variables influence this maintenance.

Our results indicate that self-reported post-treatment gains were effectively sustained for at least 12 months. Inattention, hyperactivity/impulsivity, and total ADHD symptoms, as well as global functioning, were significant improved in treated compared with control groups, as reported by participants, with medium-to-large ES estimates. Improvements in CGI measure were also maintained. These results support the validity of those obtained in previous post-treatment meta-analyses (Cairncross and Miller, 2016; Jensen et al., 2016; Young et al., 2016; Knouse et al., 2017). Nevertheless, according to blind assessors, between-group improvements in ADHD symptoms reported by previous meta-analytic reviews did not persevere over time.

On the other hand, the results provide further empirical support for the within-subject improvement. This study found that the post-treatment ES estimates on within-subject CGI, total ADHD symptoms, and inattention reported by Knouse et al. (2017) remained large at follow-up, for both blinded and self-reported measures, while the ES on global functioning continued to be moderate-to-large. One interesting finding is that the self-reported within-subject treatment effects on hyperactivity/impulsivity symptoms increased from moderate-to-large to large at follow-up.

Concerning the comparison between the different therapeutic options, DBT and Biofeedback are not as effective as CBT on the key outcome measures. Although MBCT reached a large ES on all ADHD symptom outcomes, evidence came only from one study. In addition, when CBT studies were isolated, the ES estimates for between-groups blind assessor-rated total ADHD symptoms became significant (SMD = 0.76; 95% CI [0.45–1.06]) and those for CGI increased to moderate-to-large (SMD = 0.72; 95% CI [0.44–0.99]). Furthermore, the majority of studies for which data were included for most outcomes used CBT. All these findings suggest that CBT could be the psychosocial intervention with the most long-term empirical support for the treatment of ADHD in adults. CBT is based on the premise that underlying neurobiological impairments hinder adults with ADHD from acquiring and using adaptive compensatory strategies (i.e., use of higher-level organization and planning strategies), which maintains and exacerbates the core symptoms and further contributes to a chronic functional impairment persisting since childhood (Knouse and Safren, 2010). That impairment, together with a negative social feedback, can lead to the development of maladaptive negative cognitions and beliefs that decrease motivation and increase avoidance behavior and mood disturbance, thus reinforcing the cycle. This CBT model of ADHD was supported by recent research findings, which found that adult ADHD is significantly related to dysfunctional cognitions, cognitive distortions and maladaptive coping strategies of escape-avoidance (Mitchell et al., 2013; Torrente et al., 2014; Strohmeier et al., 2016). Thus, CBT is aimed at the acquisition and especially the maintenance of compensatory skills, and also at the development of cognitive strategies to challenge the cognitive distortions, so that core neurobiological deficits do not translate as frequently into functional impairments (Safren et al., 2004). Therefore, a possible explanation for the stability of the improvements found on the CBT subset of studies might be that individuals with ADHD were able to learn and integrate the compensatory behavioral skills and the cognitive strategies into daily life, so that the changes were sustained throughout the time, despite the fact that treatment had ended.

Another notable finding from the moderator analyses was that, when compared, the individual setting was more effective than group treatment on the main outcome measures. This result could be explained by the fact that individual treatment is better suited to the specific needs of each individual, and that probably each participant received more attention from the therapist than in a group setting, which might increase the effectiveness of the treatment.

With respect to the source of the measures, on the one hand, only self-reported ADHD core symptom outcomes showed improvements, while blinded assessment did not, when treatment groups were compared to control groups. This finding could be an indicator of the presence of a significant placebo effect on control groups, which obtained a significant ES on the main outcome measures. On the other hand, some other significant results supporting blind-reported efficacy were obtained. First, CGI is a blinded measure, and between-groups treatment effect was small-to-moderate as measured by this instrument, while within-subject change was large. Second, a large effect was reached on within-subject blinded ADHD symptom outcomes. Third, moderator analyses only detected significant differences on the basis of the measure source (blinded vs. self-reported) on the within-subject inattention outcome, while the other comparisons were not significant. Forth, several individual studies found a significant long-term effect as reported by blind assessors (Safren et al., 2010; Emilsson et al., 2011; Young et al., 2015).

Meta-regression results indicate that follow-up length (from 3 to 12 months) does not predict treatment efficacy, which further supports the stability of the gains. In addition, the results indicate that medication is a factor that influences treatment effectiveness, according to the CGI measure, which increased when the percentage of medicated participants was greater, supporting the conclusions of several previous RCTs (Philipsen et al., 2015; Cherkasova et al., 2016). This finding offers empirical support for the combination of psychotherapy, particularly CBT, and pharmacotherapy as the most effective treatment option for adults with ADHD.

Studies using DBT, in a group setting, with active control-matching, and that were rated with an unclear risk of bias, achieved significantly lower ES in the majority of outcomes. This finding could be caused mainly by the study by Philipsen et al. (2015), which had considerable weight in the meta-analyses due to its large sample size since it was present in all these subgroups.

Thus, our findings indicate that there is self-reported evidence that the psychosocial interventions, particularly CBT in an individual setting, specifically improve ADHD core symptoms and global functioning until at least a year after the end of treatment, in comparison with control groups. Additionally, within-subject improvements are also maintained, even according to blind evaluators. These long-term gains further support the usefulness of psychosocial treatments for addressing adult ADHD. Nevertheless, our results must be interpreted with caution because of the high heterogeneity observed in the majority of the outcomes.

### Limitations and future research

The limitations of the present review require consideration to ensure appropriate interpretation of the findings. First, the statistical power and moderator analyses are limited by the small number of available studies. Second, a high risk of bias was determined for many of the outcomes. Self-reported outcome measures, the use of unblinded outcome assessors, lack of accuracy in the description of dropouts (and the reasons for these), and allocation/randomization processes were the main sources of bias. Third, we identified indicators of publication bias for some outcomes. The results of the Egger test were significant for all between-group and blind assessor-rated within-subject outcomes, and the trim and fill method reduced the confidence intervals of ES estimates for self-reported between-groups outcomes to below zero. Nevertheless, the capacity to detect bias is limited when meta-analyses are based on a limited number of small trials; therefore, the results from these analyses should be treated with considerable caution (Egger et al., 1997). For example, when there are fewer than 10 studies the power of Egger's test is insufficient to distinguish chance from genuine asymmetry (Higgins and Green, 2011). Fourth, the generalization of the findings is limited by the high levels of heterogeneity among the studies included. Fifth, the small number of MBCT and biofeedback studies included in this review (only one of each type of study) limits the interpretation of the results concerning these therapies. Sixth, the high level of attrition observed (36.6%) is a concern, since greater than 20% loss potentially threatens study validity (Schulz and Grimes, 2002). Seventh, it should be noted that the results of the moderator analyses must be interpreted with extreme caution because of the small sample size.

The use of larger samples, multi-center studies, specification of the pharmacological condition, inclusion of a proper comparison group (if possible an active control group), use of independent blinded outcome sources, accurate detailing of attrition (with intent-to-treat analyses) and the allocation/randomization process, and evaluation of maintenance of long-term gains should be addressed by future research to improve the internal validity of findings.

Furthermore, some relevant questions remain unresolved. To improve the efficiency of treatment, further research should be carried out to establish the specific weight of each therapy component in overall efficacy (e.g., organizing and planning skills, skills to reduce distractibility, cognitive restructuring, behavior analysis, and impulse control, among others). Given the heterogeneous nature of ADHD, it will be important to determine whether different psychosocial interventions (or therapy ingredients) may be specifically effective for each of its symptom dimensions (e.g., inattention, hyperactivity, and impulsivity). In addition, future research could explore the characteristics of adults with ADHD who are more likely to respond to CBT therapies. Previous studies have found that adding a psychosocial intervention improves the effects of pharmacological treatment (Safren et al., 2005; Emilsson et al., 2011; Young et al., 2015); however, more research is needed to determine whether the opposite is true. While some studies have found that adding medication to psychological therapy did not significantly improve outcomes (Weiss et al., 2012), others concluded that a combination of methylphenidate and psychotherapy is significantly more effective than psychotherapy alone (Philipsen et al., 2015; Cherkasova et al., 2016). Although it requires further investigation, some studies have demonstrated the preliminary efficacy and feasibility of the use of CBT alone (without medication) (Ramsay and Rostain, 2011; Ramsay, 2012), which would be especially useful in those, not uncommon, cases in which medication is not tolerated or is ineffective. Treatment guidelines state that psychological interventions without medication may be effective for some adults with moderate impairment; however, there are insufficient data to support this recommendation (National Institute for Health and Care Excellence, 2008).

## Conclusions

Psychosocial treatments are effective for treatment of ADHD at the end of the intervention and the gains are sustained for up to 12 months. In the long term, psychosocial interventions, particularly CBT, are effective in improving self-rated inattention, hyperactivity/impulsivity, and total ADHD symptoms, together with CGI and global functioning, in comparisons with control groups. Within-subject improvements were also significant when rated by blind assessors. However, a careful interpretation of these data is necessary because of the high level of heterogeneity and high risk of bias determined for many of the outcomes. There remain many important questions to be addressed by future studies; however, the evidence from this review suggests that psychological interventions are highly valuable and stable clinical tools for the treatment of adults with ADHD.

## Author contributions

PhD Student CL-P co-designed the study, conducted the literature searches, the study selection, the data extraction and the statistical analyses, and wrote the first draft of the manuscript. SM-S co-designed the study and conducted the study selection. JF-C co-designed study and co-wrote the first draft of the manuscript. JS-M supervised and improved the statistical analyses, and co-wrote the methods section. All authors reviewed and edited the manuscript for accuracy, and approved the final version of the manuscript.

### Conflict of interest statement

The authors declare that the research was conducted in the absence of any commercial or financial relationships that could be construed as a potential conflict of interest.

## Supplementary material

Supplementary Figure 1 Effect size index formula for between-group outcomes.

Supplementary Figure 2 Effect size index formula for within-subject outcomes.
