# Supplementary figures and images for: Long-Term Efficacy of Psychosocial Treatments for Adults With Attention-Deficit/Hyperactivity Disorder: A Meta-Analytic Review
Source: Front Psychol. 2018 May 4;9:638. doi: 10.3389/fpsyg.2018.00638 (PMC5946687; doi:10.3389/fpsyg.2018.00638)

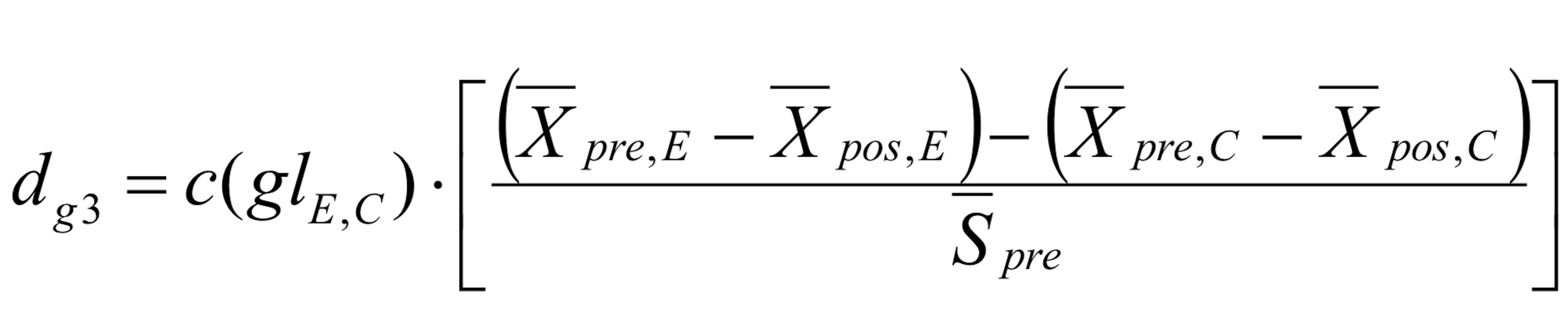

Supplement: Supplementary Figure 1 — Effect size index formula for between-group outcomes. [file Image_1.png]

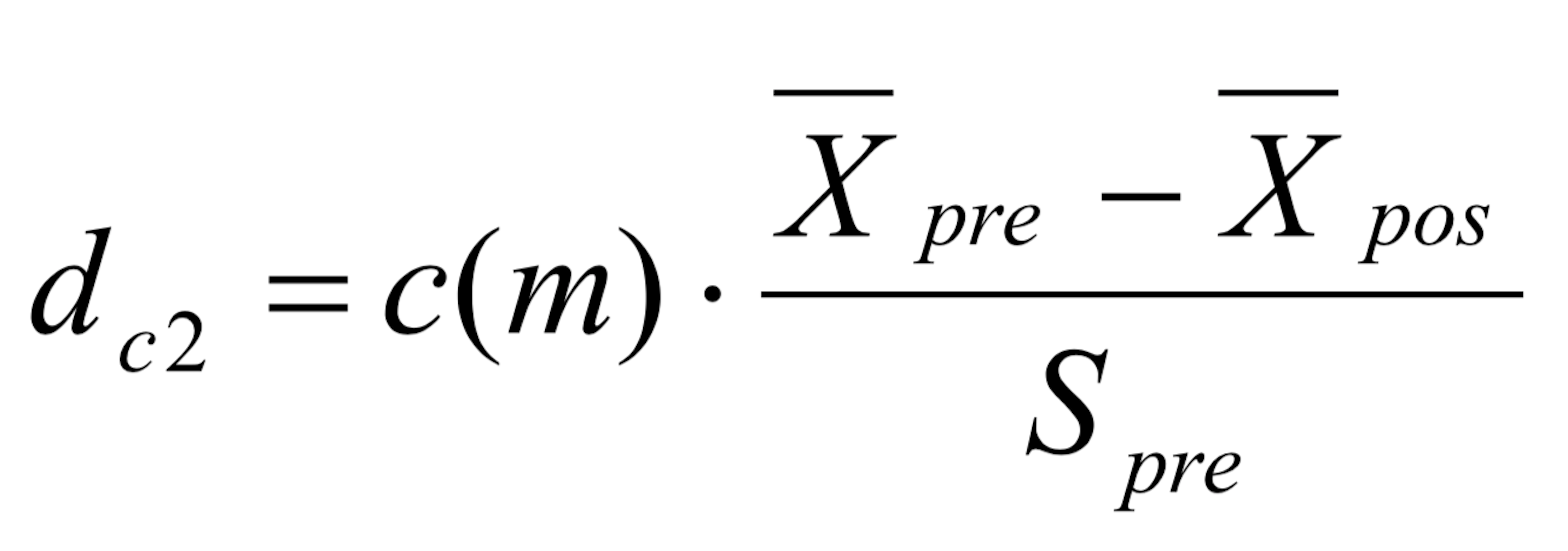

Supplement: Supplementary Figure 2 — Effect size index formula for within-subject outcomes. [file Image_2.png]
