# Supplementary material for: Long-Term Efficacy of Psychosocial Treatments for Adults With Attention-Deficit/Hyperactivity Disorder: A Meta-Analytic Review
Source: Front Psychol. 2018 May 4;9:638. doi: 10.3389/fpsyg.2018.00638 (PMC5946687; doi:10.3389/fpsyg.2018.00638)
Supplement: Supplementary file 3 [file Table_1.DOCX]

Supplementary Material

Long-term Efficacy of Psychosocial Treatments for Adults with Attention-Deficit/Hyperactivity Disorder: A Meta-Analytic Review

**Carlos López-Pinar^*^, Sonia Martínez-Sanchís, Enrique Carbonell-Vayá, Javier Fenollar-Cortés, Julio Sánchez-Meca**

*** Correspondence:**

Carlos López-Pinar

[carlopi@alumni.uv.es](mailto:carlopi@alumni.uv.es)

| **Supplementary table 1.**  Prisma 2009 checklist. | | | |
| --- | --- | --- | --- |
| Section | Topic | Item# | Reported on page |
| Title | Title | 1 | 1 |
| Abstract | Structured summary | 2 | 2 |
| Introduction | Rationale | 3 | 3-4 |
|  | Objectives | 4 | 4 |
| Methods | Protocol and registration | 5 | 5 |
|  | Eligibility criteria | 6 | 4-5 |
|  | Information sources | 7 | 5 |
|  | Search | 8 | 5 (and Supplementary Table 2) |
|  | Study selection | 9 | 6 |
|  | Data collection process | 10 | 6 |
|  | Data items | 11 | 6 |
|  | Risk of bias in individual studies | 12 | 7 |
|  | Summary measures | 13 | 6-7 |
|  | Synthesis of results | 14 | 6-7 |
|  | Risk of bias across studies | 15 | 7 |
|  | Additional analyses | 16 | 7 |
| Results | Study selection | 17 | 7-8 (and Figure 1) |
|  | Study characteristics | 18 | 7-8 (and Table 2) |
|  | Risk of bias within studies | 19 | 9 (and Supplementary Figures 11-15) |
|  | Results of individual studies | 20 | Figures 2-5 and Supplementary Figures 3-10 |
|  | Synthesis of results | 21 | 8-9 (and Figures 2-5 and Supplementary Figures 3-10) |
|  | Risk of bias across studies | 22 | 9 (and Table 3 and Supplementary Figures 11-15) |
|  | Additional analysis | 23 | 9-10 (and Supplementary Tables 4-9) |
| Discussion | Summary of evidence | 24 | 10-11 |
|  | Limitations | 25 | 11-12 |
|  | Conclusions | 26 | 12 |
| Funding | Funding | 27 | 13 |
