# Supplementary material for: Long-Term Efficacy of Psychosocial Treatments for Adults With Attention-Deficit/Hyperactivity Disorder: A Meta-Analytic Review
Source: Front Psychol. 2018 May 4;9:638. doi: 10.3389/fpsyg.2018.00638 (PMC5946687; doi:10.3389/fpsyg.2018.00638)
Supplement: Supplementary file 4 [file Table_2.DOCX]

Supplementary Material

Long-term Efficacy of Psychosocial Treatments for Adults with Attention-Deficit/Hyperactivity Disorder: A Meta-Analytic Review

**Carlos López-Pinar^*^, Sonia Martínez-Sanchís, Enrique Carbonell-Vayá, Javier Fenollar-Cortés, Julio Sánchez-Meca**

*** Correspondence:**

Carlos López-Pinar

[carlopi@alumni.uv.es](mailto:carlopi@alumni.uv.es)

| **Supplementary table 2.**  Search terms used. | | |
| --- | --- | --- |
| Search Area | Search terms | |
|  | MedLine (via PubMed) | Scopus |
| Participants: adults | (adult[Title/Abstract] OR adults[Title/Abstract]) | (TITLE-ABS-KEY(Adult) OR TITLE-ABS-KEY(adults)) |
| Intervention: Psychosocial treatments | (Psychosocial treatment[Title/Abstract] OR Skills training[Title/Abstract] OR CBT[Title/Abstract] OR Cognitive Behavioral Therapy[Title/Abstract] OR DBT[Title/Abstract] OR Dialectical Behavior Therapy[Title/Abstract] OR Neurofeedback[Title/Abstract] OR MBCT[Title/Abstract] OR Mindfulness[Title/Abstract]) | TITLE-ABS-KEY(psychosocial treatment) OR TITLE-ABS-KEY(CBT) OR TITLE-ABS-KEY(cognitive behavioral therapy) OR TITLE-ABS-KEY(dbt) OR TITLE-ABS-KEY(dialectical behavior therapy) OR TITLE-ABS-KEY(neurofeedback) OR TITLE-ABS-KEY(mindfulness) OR TITLE-ABS-KEY(MBCT) |
| Outcome: Attention deficit/hyperactivity disorder symptoms | (adhd[Title/Abstract] OR Attention Deficit Hyperactivity Disorder[Title/Abstract] OR Attention Deficit Disorder[Title/Abstract]) | (TITLE-ABS-KEY(ADHD) OR TITLE-ABS-KEY(Attention Deficit Hyperactivity Disorder) OR TITLE-ABS-KEY(Attention Deficit Disorder) |
| Language: English | *( English[lang])* | ( LIMIT-TO(LANGUAGE,"English" ) |
| Limits | Humans[Mesh] | ( LIMIT-TO(DOCTYPE,"ar" ) ) AND ( LIMIT-TO(SUBJAREA,"MEDI" ) OR LIMIT-TO(SUBJAREA,"PSYC" ) OR LIMIT-TO(SUBJAREA,"NEUR" ) |
