# Supplementary material for: Long-Term Efficacy of Psychosocial Treatments for Adults With Attention-Deficit/Hyperactivity Disorder: A Meta-Analytic Review
Source: Front Psychol. 2018 May 4;9:638. doi: 10.3389/fpsyg.2018.00638 (PMC5946687; doi:10.3389/fpsyg.2018.00638)
Supplement: Supplementary file 5 [file Table_3.DOCX]

Supplementary Material

Long-term Efficacy of Psychosocial Treatments for Adults with Attention-Deficit/Hyperactivity Disorder: A Meta-Analytic Review

**Carlos López-Pinar^*^, Sonia Martínez-Sanchís, Enrique Carbonell-Vayá, Javier Fenollar-Cortés, Julio Sánchez-Meca**

*** Correspondence:**

Carlos López-Pinar

[carlopi@alumni.uv.es](mailto:carlopi@alumni.uv.es)

| **Supplementary table 3.**  Excluded studies list and failed criteria. | |
| --- | --- |
| Reference | Failed criteria |
| Anastopoulos et al., 2015 | Does not include a follow-up assessment. |
| Barth et al., 2017 | Does not include a follow-up assessment. |
| Bihlar Muld et al., 2016 | Participants had severe substance abuse; Does not include a follow-up assessment. |
| Bramham et al., 2009 | ADHD symptoms valid measure was not included; Allocation is not randomized; Does not include a follow-up assessment. |
| Bueno et al., 2015 | Allocation is not randomized; Does not include a follow-up assessment. |
| Cherkasova et al., 2016^a^ | All groups received a specific for ADHD psychosocial treatment. |
| Cowley et al., 2016 | Does not include a follow-up assessment. |
| Edel et al., 2013 | All groups received a specific for ADHD psychosocial treatment; Does not include a follow-up assessment. |
| Hepark et al., 2014 | Does not include a follow-up assessment. |
| Hepark et al., 2015 | Does not include a follow-up assessment. |
| Hesslinger et al., 2002 | Does not include a follow-up assessment. |
| Hiltunen et al., 2014 | All groups received a specific for ADHD psychosocial treatment. |
| Hirvikoski et al., 2011 | Does not include a follow-up assessment. |
| Hirvikoski et al., 2015 | ADHD symptoms valid measure was not included; Does not include a follow-up assessment. |
| Kaiser, 1997 | ADHD symptoms valid measure was not included; Not published on peer-reviewed journal; Does not include a follow-up assessment. |
| LaCount et al., 2015 | Does not include a follow-up assessment. |
| Mayer et al., 2012 | Insufficient data showed on article; Does not include a follow-up assessment. |
| Mitchell et al., 2013 | Does not include a follow-up assessment. |
| Moëll et al., 2015 | Treatment was self-applied; Does not include a follow-up assessment. |
| Pettersson et al., 2014 (iCBT-S group) | Treatment was self-applied. |
| Philipsen et al., 2007 | Does not include a follow-up assessment. |
| Prada et al., 2015 | Participants had a personality disorder; Does not include a follow-up assessment. |
| Ramsay & Rostain, 2011 | Does not include a follow-up assessment. |
| Rostain & Ramsay, 2006 | Does not include a follow-up assessment. |
| Safren et al., 2005 | Does not include a follow-up assessment. |
| Schoenberg et al., 2014 | Does not include a follow-up assessment. |
| Solanto et al., 2008 | Does not include a follow-up assessment. |
| Solanto et al., 2010 | Does not include a follow-up assessment. |
| Stern et al., 2016 | Does not include a follow-up assessment. |
| Stevenson et al., 2002 | Does not include a follow-up assessment. |
| Stevenson et al., 2003 | Data showed in article is wrong (same data is the same as in Stevenson et al., 2002); Does not include a follow-up assessment. |
| Thompson et al., 1998 | ADHD symptoms valid measure was not included; Does not include a follow-up assessment. |
| Vidal-Estrada et al., 2013 | All groups received a specific for ADHD psychosocial treatment; Does not include a follow-up assessment. |
| Vidal-Estrada et al., 2015 | Not all participants were older than 18; Does not include a follow-up assessment. |
| Virta et al., 2008 | Does not include a follow-up assessment. |
| Virta et al., 2010 | Does not include a follow-up assessment. |
| Virta et al., 2010a | Does not include a follow-up assessment. |
| Virta et al., 2015 | ADHD symptoms valid measure was not included; Allocation is not randomized; Does not include a follow-up assessment. |
| Wang et al., 2016 | Does not include a follow-up assessment. |
| Weiss et al., 2006 | All groups received a specific for ADHD psychosocial treatment; Does not include a follow-up assessment. |
| Weiss et al., 2012 | All groups received a specific for ADHD psychosocial treatment; Does not include a follow-up assessment. |
| White et al., 2006 | All groups received a specific for ADHD psychosocial treatment; Does not include a follow-up assessment. |
| Wiggins et al., 1999 | Allocation is not randomized; Does not include a follow-up assessment. |
| Wilens et al., 1999 | Study is a review of several cases; Therapy setting is different in every case; Does not include a follow-up assessment. |
| Wymbs et al., 2015 | Formal diagnostic assessment for ADHD was not made; Does not include a follow-up assessment. |
| Zilverstand et al., 2017 | Does not include a follow-up assessment. |
| Zwart et al., 2001 | ADHD symptoms valid measure was not included; treatment used was not a proper psychological therapy; Does not include a follow-up assessment. |
| Zylowska et al., 2008 | Not all participants were older than 18; adults and adolescent participants were mixed at ADHD symptoms outcome; 4 subjects without a formal ADHD diagnostic were included; Does not include a follow-up assessment. |
| ^a^CBT ALONE and CBT+M were included in within-subjects meta-analyses | |

**References**

Anastopoulos, A. D., & King, K. A. (2015). A Cognitive-Behavior Therapy and Mentoring Program for College Students With ADHD. *Cognitive and Behavioral Practice*, *22*(2), 141–151. https://doi.org/10.1016/j.cbpra.2014.01.002

Barth, B., Mayer, K., Strehl, U., Fallgatter, A. J., & Ehlis, A. C. (2017). EMG biofeedback training in adult attention-deficit/hyperactivity disorder: An active (control) training? *Behavioural Brain Research*, *329*(January), 58–66. https://doi.org/10.1016/j.bbr.2017.04.021

Bihlar Muld, B., Jokinen, J., Bölte, S., & Hirvikoski, T. (2016). Skills training groups for men with ADHD in compulsory care due to substance use disorder: a feasibility study. *ADHD Attention Deficit and Hyperactivity Disorders*, *8*(3), 159–172. https://doi.org/10.1007/s12402-016-0195-4

Bramham, J., Young, S., Bickerdike, A., Spain, D., McCartan, D., & Xenitidis, K. (2009). Evaluation of group cognitive behavioral therapy for adults with ADHD. *Journal of Attention Disorders*, *12*(5), 434–41. https://doi.org/10.1177/1087054708314596

Bueno, V. F., Kozasa, E. H., Aparecida, M., Alves, T. M., Louzã, M. R., & Pompéia, S. (2015). Mindfulness Meditation Improves Mood , Quality of Life , and Attention in Adults with Attention Deficit Hyperactivity Disorder. *BioMed Research International*, *2015*, 1–14. Retrieved from http://www.ncbi.nlm.nih.gov/pubmed/19346466

Cherkasova, M. V., French, L. R., Syer, C. A., Cousins, L., Galina, H., Ahmadi-Kashani, Y., & Hechtman, L. (2016). Efficacy of Cognitive Behavioral Therapy With and Without Medication for Adults With ADHD: A Randomized Clinical Trial. *Journal of Attention Disorders*. https://doi.org/10.1177/1087054716671197

Cowley, B., Holmström, É., Juurmaa, K., Kovarskis, L., & Krause, C. M. (2016). Computer Enabled Neuroplasticity Treatment: A Clinical Trial of a Novel Design for Neurofeedback Therapy in Adult ADHD. *Frontiers in Human Neuroscience*, *10*(205), 1–13. https://doi.org/10.3389/fnhum.2016.00205

Edel, M.-A., Hölter, T., Wassink, K., & Juckel, G. (2013). A Comparison of Mindfulness-Based Group Training and Skills Group Training in Adults With ADHD: An Open Study. *Journal of Attention Disorders*, 1–11. https://doi.org/10.1177/1087054714551635

Hepark, S., Janssen, L., de Vries, A., Schoenberg, P. L. A., Donders, R., Kan, C. C., & Speckens, A. E. M. (2015). The Efficacy of Adapted MBCT on Core Symptoms and Executive Functioning in Adults With ADHD: A Preliminary Randomized Controlled Trial. *Journal of Attention Disorders*, 1087054715613587. https://doi.org/10.1177/1087054715613587

Hepark, S., Kan, C. C., & Speckens, A. (2014). Toepasbaarheid en effectiviteit van mindfulnesstraining bij volwassenen met AD(H)D; een open pilotonderzoek. *Tijdschrift Voor Psychiatrie*, *56*(7), 471–476.

Hesslinger, B., Tebartz Van Elst, L., Nyberg, E., Dykierek, P., Richter, H., Berner, M., & Ebert, D. (2002). Psychotherapy of attention deficit hyperactivity disorder in adults. *Eur Arch Psychiatry Clin Neurosci*, *252*, 177–184. https://doi.org/10.1007/s00406-002-0379-0

Hiltunen, S., Virta, M., Salakari, A., Antila, M., Chydenius, E., Kaski, M., … Partinen, M. (2014). Better long-term outcome for hypnotherapy than for CBT in adults with ADHD: Results of a six-month follow-up. *Contemporary Hypnosis and Integrative Therapy*, *30*(3), 118–134.

Hirvikoski, T., Waaler, E., Alfredsson, J., Pihlgren, C., Holmström, A., Johnson, A., … Nordström, A. L. (2011). Reduced ADHD symptoms in adults with ADHD after structured skills training group: Results from a randomized controlled trial. *Behaviour Research and Therapy*, *49*(3), 175–185. https://doi.org/10.1016/j.brat.2011.01.001

Hirvikoski, T., Waaler, E., Lindström, T., Bölte, S., & Jokinen, J. (2015). Cognitive behavior therapy-based psychoeducational groups for adults with ADHD and their significant others (PEGASUS): an open clinical feasibility trial. *ADHD Attention Deficit and Hyperactivity Disorders*, *7*(1), 89–99. https://doi.org/10.1007/s12402-014-0141-2

Kaiser, D. A. (1997). Efficacy of Neurofeedback on Adults with Attentional Deficit and Related Disorders. *EEG Spectrum*, 1–6. Retrieved from http://www.eegspectrum.com/Applications/ADHD-ADD/AdultsAttDefRelDis- Intro/

LaCount, P. A., Hartung, C. M., Shelton, C. R., Clapp, J. D., & Clapp, T. K. W. (2015). Preliminary Evaluation of a Combined Group and Individual Treatment for College Students With Attention-Deficit/Hyperactivity Disorder. *Cognitive and Behavioral Practice*, *22*(2), 152–160. https://doi.org/10.1016/j.cbpra.2014.07.004

Mayer, K., Wyckoff, S. N., Schulz, U., & Strehl, U. (2012). Neurofeedback for Adult Attention-Deficit/Hyperactivity Disorder: Investigation of Slow Cortical Potential Neurofeedback—Preliminary Results. *Journal of Neurotherapy*, *16*(1), 37–45. https://doi.org/10.1080/10874208.2012.650113

Mitchell, J. T., McIntyre, E. M., English, J. S., Dennis, M. F., Beckham, J. C., & Kollins, S. H. (2013). A Pilot Trial of Mindfulness Meditation Training for ADHD in Adulthood: Impact on Core Symptoms, Executive Functioning, and Emotion Dysregulation. *Journal of Attention Disorders*, 1087054713513328-. https://doi.org/10.1177/1087054713513328

Moëll, B., Kollberg, L., Nasri, B., Lindefors, N., & Kaldo, V. (2015). Living smart - a randomized controlled trial of a guided online course teaching adults with adhd or sub-clinical adhd to use smartphones to structure their everyday life. *Internet Interventions*, *2*(1), 24–31. https://doi.org/10.1016/j.invent.2014.11.004

Pettersson, R., Söderström, S., Edlund-Söderström, K., & Nilsson, K. W. (2014). Internet-Based Cognitive Behavioral Therapy for Adults With ADHD in Outpatient Psychiatric Care: A Randomized Trial. *Journal of Attention Disorders*, 1087054714539998-. https://doi.org/10.1177/1087054714539998

Philipsen, A., Richter, H., Peters, J., Alm, B., Sobanski, E., Colla, M., … Hesslinger, B. (2007). Structured Group Psychotherapy in Adults With Attention Deficit Hyperactivity Disorder. *The Journal of Nervous and Mental Disease*, *195*(12), 1013–1019. https://doi.org/10.1097/NMD.0b013e31815c088b

Prada, P., Nicastro, R., Zimmermann, J., Hasler, R., Aubry, J. M., & Perroud, N. (2015). Addition of methylphenidate to intensive dialectical behaviour therapy for patients suffering from comorbid borderline personality disorder and ADHD: a naturalistic study. *ADHD Attention Deficit and Hyperactivity Disorders*, *7*(3), 199–209. https://doi.org/10.1007/s12402-015-0165-2

Ramsay, J. R., & Rostain, A. L. (2011). CBT Without Medications for Adult ADHD: An Open Pilot Study of Five Patients. *Journal of Cognitive Psychotherapy: An International Quarterly*, *25*(4), 277–286. https://doi.org/http://dx.doi.org/10.1891/0889-8391.25.4.277

Rostain, A. L., & Ramsay, J. R. (2006). A combined treatment approach for adults with ADHD--results of an open study of 43 patients. *Journal of Attention Disorders*, *10*(2), 150–9. https://doi.org/10.1177/1087054706288110

Safren, S. A., Otto, M. W., Sprich, S., Winett, C. L., Wilens, T. E., & Biederman, J. (2005). Cognitive-behavioral therapy for ADHD in medication-treated adults with continued symptoms. *Behaviour Research and Therapy*, *43*(7), 831–842. https://doi.org/10.1016/j.brat.2004.07.001

Schoenberg, P. L. A., Hepark, S., Kan, C. C., Barendregt, H. P., Buitelaar, J. K., & Speckens, A. E. M. (2014). Effects of mindfulness-based cognitive therapy on neurophysiological correlates of performance monitoring in adult attention-deficit/hyperactivity disorder. *Clinical Neurophysiology*, *125*(7), 1407–1416. https://doi.org/10.1016/j.clinph.2013.11.031

Solanto, M. V, Marks, D. J., Mitchell, K. J., Wasserstein, J., & Kofman, M. D. (2008). Development of a new psychosocial treatment for adult ADHD. *Journal of Attention Disorders*, *11*(6), 728–736. https://doi.org/10.1177/1087054707305100

Solanto, M. V, Marks, D. J., Wasserstein, J., Abikoff, H., Alvir, J. M. J., & Kofman, M. D. (2010). Effects of Meta-Cognitive Therapy (MCT) for Adult ADHD. *American Journal of Psychiatry*, *167*(8), 958–968. https://doi.org/10.1176/appi.ajp.2009.09081123.Efficacy

Stern, A., Malik, E., Pollak, Y., Bonne, O., & Maeir, A. (2016). The Efficacy of Computerized Cognitive Training in Adults With ADHD: A Randomized Controlled Trial. *Journal of Attention Disorders*, *20*(12), 991–1003. https://doi.org/10.1177/1087054714529815

Stevenson, C. S., Stevenson, R. J., & Whitmont, S. (2003). A self-directed psychosocial intervention with minimal therapist contact for adults with attention deficit hyperactivity disorder. *Clinical Psychology and Psychotherapy*, *10*(2), 93–101. https://doi.org/10.1002/cpp.356

Stevenson, C. S., Whitmont, S., Bornholt, L., Livesey, D., & Stevenson, R. J. (2002). A cognitive remediation programme for adults with Attention Deficit Hyperactivity Disorder. *The Australian and New Zealand Journal of Psychiatry*, *36*(5), 610–6. https://doi.org/10.1046/j.1440-1614.2002.01052.x

Thompson, L., & Thompson, M. (1998). Neurofeedback combined with training in metacognitive strategies: Effectiveness in students with ADD. *Applied Psychophysiology Biofeedback*, *23*(4), 243–263. https://doi.org/10.1023/A:1022213731956

Vidal-Estrada, R., Bosch, R., Nogueira, M., Gómez-Barros, N., Valero, S., Palomar, G., … Ramos-Quiroga, J. A. (2013). Psychoeducation for adults with attention deficit hyperactivity disorder vs. cognitive behavioral group therapy: a randomized controlled pilot study. *The Journal of Nervous and Mental Disease*, *201*(10), 894–900. https://doi.org/10.1097/NMD.0b013e3182a5c2c5

Vidal-Estrada, R., Castells, J., Richarte, V., Palomar, G., Garc??a, M., Nicolau, R., … Ramos-Quiroga, J. A. (2015). Group therapy for adolescents with attention-deficit/hyperactivity disorder: A randomized controlled trial. *Journal of the American Academy of Child and Adolescent Psychiatry*, *54*(4), 275–282. https://doi.org/10.1016/j.jaac.2014.12.016

Virta, M., Hiltunen, S., Mattsson, M., & Kallio, S. (2015). The impact of hypnotic suggestions on reaction times in continuous performance test in adults with ADHD and healthy controls. *PLoS ONE*, *10*(5), 1–15. https://doi.org/10.1371/journal.pone.0126497

Virta, M., Salakari, A., Antila, M., Chydenius, E., Partinen, M., Kaski, M., … Iivanainen, M. (2010a). Hypnotherapy for adults with attention deficit hyperactivity disorder: A randomized controlled study. *Contemporary Hypnosis*, *27*(1), 5–18.

Virta, M., Salakari, A., Antila, M., Chydenius, E., Partinen, M., Kaski, M., … Iivanainen, M. (2010b). Short cognitive behavioral therapy and cognitive training for adults with ADHD - a randomized controlled pilot study. *Neuropsychiatric Disease and Treatment*, *6*, 443–53. https://doi.org/10.3200/ENVT.50.5.12-25

Virta, M., Vedenpää, A., Grönroos, N., Chydenius, E., Partinen, M., Vataja, R., … Iivanainen, M. (2008). Adults With ADHD Benefit From Cognitive–Behaviorally Oriented Group Rehabilitation: A Study of 29 Participants. *Journal of Attention Disorders*, *12*(3), 218–226.

Wang, X., Cao, Q., Wang, J., Wu, Z., Wang, P., Sun, L., … Wang, Y. (2016). The effects of cognitive-behavioral therapy on intrinsic functional brain networks in adults with attention-deficit/hyperactivity disorder. *Behaviour Research and Therapy*, *76*, 32–39. https://doi.org/10.1016/j.brat.2015.11.003

Weiss, M., & Hechtman, L. (2006). A randomized double-blind trial of paroxetine and/or dextroamphetamine and problem-focused therapy for attention-deficit/hyperactivity disorder in adults. *Journal of Clinical Psychiatry*, *67*(4), 611–619. https://doi.org/10.4088/JCP.v67n0412

Weiss, M., Murray, C., Wasdell, M., Greenfield, B., Giles, L., & Hechtman, L. (2012). A randomized controlled trial of CBT therapy for adults with ADHD with and without medication. *BMC Psychiatry*, *12*(1), 30. https://doi.org/10.1186/1471-244X-12-30

White, H. a, & Shah, P. (2006). Training attention-switching ability in adults with ADHD. *Journal of Attention Disorders*, *10*(1), 44–53. https://doi.org/10.1177/1087054705286063

Wiggins, D., Singh, K., Getz, H. G., & Hutchins, D. E. (1999). Effects of Brief Group Intervention for Adults with Attention Deficit/Hyperactivity Disorder. *Journal of Mental Health Counseling*, *21*(1), 82–92.

Wilens, T. E., Mcdermott, S. P., & Spencer, T. J. (1999). Cognitive Therapy in the Treatment of Adults With ADHD : A Systematic Chart Review of 26 Cases. *Journal of Cognitive Psychotherapy: An International Quarterly*, *13*(3), 215–226.

Wymbs, B. T., & Molina, B. S. G. (2015). Integrative Couples Group Treatment for Emerging Adults With ADHD Symptoms. *Cognitive and Behavioral Practice*, *22*(2), 161–171. https://doi.org/10.1016/j.cbpra.2014.06.008

Zilverstand, A., Sorger, B., Slaats-Willemse, D., Kan, C. C., Goebel, R., & Buitelaar, J. K. (2017). fMRI neurofeedback training for increasing anterior cingulate cortex activation in adult attention deficit hyperactivity disorder. An exploratory randomized, single-blinded study. *PLoS ONE*, *12*(1), 1–23. https://doi.org/10.1371/journal.pone.0170795

Zwart, L. M., & Kallemeyn, L. M. (2001). Peer-Based Coaching for College Students with ADHD and Learning Disabilities Lavonne M. Zwart Leanne M. Kallemeyn Calvin College. *J Postsecondary Educ Disabil*, *15*, 1–5.

Zylowska, L., Ackerman, D. L., Yang, M. H., Futrell, J. L., Horton, N. L., Hale, T. S., & Smalley, S. L. (2008). Mindfulness Meditation Training in Adults and Adolescents With ADHD: A Feasibility Study. *Journal of Attention Disorders*, *11*(6), 737–746. https://doi.org/10.1177/1087054707308502
