# Supplementary material for: Long-Term Efficacy of Psychosocial Treatments for Adults With Attention-Deficit/Hyperactivity Disorder: A Meta-Analytic Review
Source: Front Psychol. 2018 May 4;9:638. doi: 10.3389/fpsyg.2018.00638 (PMC5946687; doi:10.3389/fpsyg.2018.00638)
Supplement: Supplementary file 6 [file Table_4.DOCX]

Supplementary Material

Long-term Efficacy of Psychosocial Treatments for Adults with Attention-Deficit/Hyperactivity Disorder: A Meta-Analytic Review

**Carlos López-Pinar^*^, Sonia Martínez-Sanchís, Enrique Carbonell-Vayá, Javier Fenollar-Cortés, Julio Sánchez-Meca**

*** Correspondence:**

Carlos López-Pinar

[carlopi@alumni.uv.es](mailto:carlopi@alumni.uv.es)

| **Supplementary Table 4.**  Summary of Cochrane Collaboration’s tool for assessing risk of bias for total ADHD symptoms outcome. | | | | | | | |
| --- | --- | --- | --- | --- | --- | --- | --- |
|  | Domain | | | | | | |
| Study | Randomiza-tion sequence | Allocation concealment | Blinding outcome assessment | Incomplete outcome | Selective reporting | Medication stabilization | **Within trial summary** |
| Cherkasova et al., 2016 | - | - | High risk | Low risk | Low risk | Low risk | **High risk** |
| Emilsson et al., 2011 | Low risk | Low risk | Low risk | Unclear risk | Low risk | Low risk | **Unclear risk** |
| Fleming et al., 2015 | Unclear risk | Unclear risk | High risk | Low risk | Low risk | Low risk | **High risk** |
| Gu et al., 2017 | Low risk | Unclear risk | High risk | Low risk | Low risk | Low risk | **High risk** |
| Morgensterns et al., 2016 | - | - | High risk | Unclear risk | Low risk | Unclear risk | **High risk** |
| Nasri et al., 2017 | - | - | High risk | Low risk | Low risk | Low risk | **High risk** |
| Pettersson et al., 2014 | - | - | High risk | Low risk | Low risk | High risk | **High risk** |
| Philipsen et al., 2015 | Low risk | Low risk | Low risk | Unclear risk | Low risk | Low risk | **Unclear risk** |
| Safren et al., 2010 | Unclear risk | Unclear risk | Low risk | Unclear risk | Low risk | Low risk | **Unclear risk** |
| Salakari et al., 2010 | - | - | High risk | Unclear risk | Unclear risk | High risk | **High risk** |
| Salomone et al., 2015 | Low risk | Low risk | High risk | High risk | Low risk | Low risk | **High risk** |
| Young et al., 2015 | Low risk | Low risk | Low risk | Low risk | Low risk | Low risk | **Low risk** |
