# Supplementary material for: Long-Term Efficacy of Psychosocial Treatments for Adults With Attention-Deficit/Hyperactivity Disorder: A Meta-Analytic Review
Source: Front Psychol. 2018 May 4;9:638. doi: 10.3389/fpsyg.2018.00638 (PMC5946687; doi:10.3389/fpsyg.2018.00638)
Supplement: Supplementary file 10 [file Table_8.DOCX]

Supplementary Material

Long-term Efficacy of Psychosocial Treatments for Adults with Attention-Deficit/Hyperactivity Disorder: A Meta-Analytic Review

**Carlos López-Pinar^*^, Sonia Martínez-Sanchís, Enrique Carbonell-Vayá, Javier Fenollar-Cortés, Julio Sánchez-Meca**

*** Correspondence:**

Carlos López-Pinar

[carlopi@alumni.uv.es](mailto:carlopi@alumni.uv.es)

| **Supplementary Table 8.**  Summary of Cochrane Collaboration’s tool for assessing risk of bias for global functioning outcome. | | | | | | | |
| --- | --- | --- | --- | --- | --- | --- | --- |
|  | Domain | | | | | | |
| Study | Randomiza-tion sequence | Allocation concealment | Blinding outcome assessment | Incomplete outcome | Selective reporting | Medication stabilization | **Within trial summary** |
| Cherkasova et al., 2016 | - | - | High risk | Low risk | Low risk | Low risk | High risk |
| Emilson et al., 2011 | Low risk | Low risk | High risk | Unclear risk | Low risk | Low risk | High risk |
| Morgensters et al., 2016 | - | - | High risk | Unclear risk | Low risk | Unclear risk | High risk |
| Petterson et al., 2014 | - | - | High risk | Low risk | Low risk | High risk | High risk |
| Young et al., 2015 | Low risk | Low risk | High risk | Low risk | Low risk | Low risk | High risk |
