# Supplementary material for: Long-Term Efficacy of Psychosocial Treatments for Adults With Attention-Deficit/Hyperactivity Disorder: A Meta-Analytic Review
Source: Front Psychol. 2018 May 4;9:638. doi: 10.3389/fpsyg.2018.00638 (PMC5946687; doi:10.3389/fpsyg.2018.00638)
Supplement: Supplementary file 11 [file Table_9.DOCX]

Supplementary Material

Long-term Efficacy of Psychosocial Treatments for Adults with Attention-Deficit/Hyperactivity Disorder: A Meta-Analytic Review

**Carlos López-Pinar^*^, Sonia Martínez-Sanchís, Enrique Carbonell-Vayá, Javier Fenollar-Cortés, Julio Sánchez-Meca**

*** Correspondence:**

Carlos López-Pinar

[carlopi@alumni.uv.es](mailto:carlopi@alumni.uv.es)

| **Supplementary Table 9.**  Publication bias analyses for between-groups outcomes. | | | | | | | | | | | |
| --- | --- | --- | --- | --- | --- | --- | --- | --- | --- | --- | --- |
|  |  |  |  |  | Egger regression test | | |  | Trim and Fill | | |
| Outcome | Rater | Studies | Fail-safe N |  | Bias | 95% CI | p value |  | Studies trimmed | Adjusted SMD | 95% CI |
| Total ADHD symptoms | Self- rated | 7 | 56 |  | 6.26 | 1.99 to 10.54 | <0.01 |  | 2 | 0.40 | -0.05 to 0.85 |
|  | Blind-assessor | 5 | 3 |  | 5.31 | -1.22 to 11.85 | 0.04 |  | 2 | 0.07 | -0.39 to 0.53 |
| Inattention symptoms | Self- rated | 7 | 50 |  | 5.17 | 2.94 to 8.40 | <0.01 |  | 3 | 0.36 | <0.01 to 0.73 |
|  | Blind-assessor | 3 | 0 |  | 6.69 | -4.87 to 18.26 | 0.04 |  | 0 | 0.16 | -0.27 to 0.59 |
| Hyperactivity/  impulsivity symptoms | Self- rated | 6 | 42 |  | 5.71 | -0.01 to 11.45 | 0.02 |  | 0 | 0.66 | 0.18 to 1.14 |
|  | Blind-assessor | 3 | 1 |  | 6.48 | -4.49 to 17.45 | 0.04 |  | 0 | 0.27 | -0.16 to 0.70 |
| CGI | Blind-assessor | 5 | 19 |  | 4 | -2.03 to 10.03 | 0.06 |  | 1 | 0.39 | 0.09 to 0.69 |
| Global functioning | Self- rated | 3 | 10 |  | 0.32 | -103.17 to 103.81 | 0.49 |  | 0 | 0.76 | 0.21 to 1.31 |
