# Supplementary material for: Long-Term Efficacy of Psychosocial Treatments for Adults With Attention-Deficit/Hyperactivity Disorder: A Meta-Analytic Review
Source: Front Psychol. 2018 May 4;9:638. doi: 10.3389/fpsyg.2018.00638 (PMC5946687; doi:10.3389/fpsyg.2018.00638)
Supplement: Supplementary file 12 [file Table_10.DOCX]

Supplementary Material

Long-term Efficacy of Psychosocial Treatments for Adults with Attention-Deficit/Hyperactivity Disorder: A Meta-Analytic Review

**Carlos López-Pinar^*^, Sonia Martínez-Sanchís, Enrique Carbonell-Vayá, Javier Fenollar-Cortés, Julio Sánchez-Meca**

*** Correspondence:**

Carlos López-Pinar

[carlopi@alumni.uv.es](mailto:carlopi@alumni.uv.es)

| **Supplementary Table 10.**  Publication bias analyses for within-subject outcomes. | | | | | | | | | | | |
| --- | --- | --- | --- | --- | --- | --- | --- | --- | --- | --- | --- |
|  |  |  |  |  | Egger regression test | | |  | Trim and Fill | | |
| Outcome | Rater | Studies | Fail-safe N |  | Bias | 95% CI | p value |  | Studies trimmed | Adjusted SMD | 95% CI |
| Total ADHD symptoms | Self- rated | 13 | 631 |  | 0.90 | -2.09 to 3.90 | 0.26 |  | 0 | 1.08 | 0.88 to 1.29 |
|  | Blind assessor | 5 | 157 |  | 2.81 | -1.18 to 6.90 | 0.06 |  | 2 | 1.14 | 0.86 to 1.42 |
| Inattention symptoms | Self- rated | 7 | 327 |  | 3.10 | -0.47 to 6.67 | 0.04 |  | 0 | 1.32 | 0.99 to 1.64 |
|  | Blind assessor | 4 | 45 |  | 1.93 | -1.55 to 5.41 | 0.04 |  | 0 | 0.95 | 0.72 to 1.19 |
| Hyperactivity/  impulsivity symptoms | Self- rated | 7 | 169 |  | 2.48 | -2.87 to 7.82 | 0.14 |  | 0 | 1.05 | 0.70 to 1.39 |
|  | Blind assessor | 3 | 42 |  | 6.90 | -3.42 to 17.38 | 0.04 |  | 0 | 0.96 | 0.50 to 1.42 |
| CGI | Blind assessor | 5 | 130 |  | 0.24 | -4.39 to 4.87 | 0.44 |  | 0 | 1.16 | 0.95 to 1.37 |
| Global functioning | Self- rated | 4 | 16 |  | 3.35 | -10.39 to 17.09 | 0.20 |  | 1 | 0.54 | 0.05 to 1.03 |
