# Supplementary material for: Long-Term Efficacy of Psychosocial Treatments for Adults With Attention-Deficit/Hyperactivity Disorder: A Meta-Analytic Review
Source: Front Psychol. 2018 May 4;9:638. doi: 10.3389/fpsyg.2018.00638 (PMC5946687; doi:10.3389/fpsyg.2018.00638)
Supplement: Supplementary file 13 [file Table_11.DOCX]

Supplementary Material

Long-term Efficacy of Psychosocial Treatments for Adults with Attention-Deficit/Hyperactivity Disorder: A Meta-Analytic Review

**Carlos López-Pinar^*^, Sonia Martínez-Sanchís, Enrique Carbonell-Vayá, Javier Fenollar-Cortés, Julio Sánchez-Meca**

*** Correspondence:**

Carlos López-Pinar

[carlopi@alumni.uv.es](mailto:carlopi@alumni.uv.es)

| Supplementary Table 11.  Subgroup analyses for between-groups outcomes. | | | | | | | |
| --- | --- | --- | --- | --- | --- | --- | --- |
| Outcome | Moderator variable | χ^2^ test | *p* value | Subgroup | *k*  studies | SMD | 95% CI |
| Total ADHD symptoms | Risk of bias | 11.74 | <0.01 | Low | 1 | 0.99 | -0.50 to 1.48 |
|  |  |  |  | Unclear | 4 | 0.23 | -0.19 to -0.66 |
|  |  |  |  | High | 3 | 1.21 | 0.83 to 1.59 |
|  | Therapy | 16.47 | <0.01 | CBT | 3 | 0.76 | 0.45 to 1.06 |
|  |  |  |  | DBT | 3 | 0.14 | -0.33 to 0.60 |
|  |  |  |  | MBCT | 1 | 1.48 | 0.97 to 1.99 |
|  |  |  |  | BFB | 1 | 1.26 | 0.59 to 1.93 |
|  | Treatment setting | 36.68 | <0.01 | Individual | 3 | 1.07 | 0.43 to 1.70 |
|  |  |  |  | Combined | 3 | 0.94 | 0.60 to 1.27 |
|  |  |  |  | Group | 2 | -0.12 | -0.32 to 0.07 |
|  | Outcome source | 0.85 | 0.36 | Self-report | 8 | 0.71 | 0.22 to 1.21 |
|  |  |  |  | Blind assessors | 5 | 0.40 | -0.06 to 0.85 |
|  | Control group | 5.71 | 0.06 | Active control | 4 | 0.33 | -0.17 to 0.82 |
|  |  |  |  | TAU | 2 | 0.98 | 0.57 to 1.39 |
|  |  |  |  | Waiting list | 2 | 1.18 | 0.56 to 1.79 |
| Inattention symptoms | Risk of bias | 31.22 | <0.01 | Low | 1 | 0.68 | 0.21 to 1.15 |
|  |  |  |  | Unclear | 2 | -0.08 | -0.34 to 0.19 |
|  |  |  |  | High | 4 | 1 | 0.72 to 1.28 |
|  | Therapy | 8 | 0.05 | CBT | 2 | 0.75 | 0.38 to 1.12 |
|  |  |  |  | DBT | 3 | 0.21 | -0.30 to 0.73 |
|  |  |  |  | MBCT | 1 | 1.22 | 0.75 to 1.69 |
|  |  |  |  | BFB | 1 | 0.80 | 0.19 to 1.41 |
|  | Treatment setting | 29.21 | <0.01 | Individual | 2 | 1.06 | 0.65 to 1.46 |
|  |  |  |  | Combined | 3 | 0.82 | 0.50 to 1.13 |
|  |  |  |  | Group | 2 | -0.08 | -0.34 to 0.19 |
|  | Outcome source | 2.54 | 0.11 | Self-report | 7 | 0.64 | 0.21 to 1.07 |
|  |  |  |  | Blind assessors | 3 | 0.14 | -0.29 to 0.58 |
|  | Control group | 11.61 | <0.01 | Active control | 3 | 0.14 | -0.30 to 0.58 |
|  |  |  |  | TAU | 2 | 0.75 | 0.38 to 1.12 |
|  |  |  |  | Waiting list | 2 | 1.14 | 0.77 to 1.51 |
| Hyperactivity/ impulsivity symptoms | Risk of bias | 31.40 | <0.01 | Low | 1 | 0.87 | 0.38 to 1.36 |
|  |  |  |  | Unclear | 2 | 0.06 | -0.13 to 0.26 |
|  |  |  |  | High | 3 | 1.06 | 0.74 to 1.39 |
|  | Therapy | 32.96 | <0.01 | CBT | 2 | 0.93 | 0.55 to 1.31 |
|  |  |  |  | DBT | 2 | 0.06 | -0.13 to 0.26 |
|  |  |  |  | MBCT | 1 | 1.28 | 0.79 to 1.77 |
|  |  |  |  | BFB | 1 | 0.76 | 0.15 to 1.37 |
|  | Treatment setting | 24.83 | <0.01 | Individual | 2 | 1.05 | 0.55 to 1.56 |
|  |  |  |  | Combined | 2 | 0.93 | 0.55 to 1.31 |
|  |  |  |  | Group | 2 | 0.06 | -0.13 to 0.26 |
|  | Outcome source | 1.60 | 0.21 | Self-report | 6 | 0.69 | 0.22 to 1.16 |
|  |  |  |  | Blind assessors | 3 | 0.28 | -0.13 to 0.70 |
|  | Control group | 16.46 | <0.01 | Active control | 3 | 0.20 | -0.12 to 1.51 |
|  |  |  |  | TAU | 2 | 0.93 | 0.55 to 1.31 |
|  |  |  |  | Waiting list | 1 | 1.28 | 0.79 to 1.77 |
| CGI | Risk of bias | 1.25 | 0.26 | Low | 1 | 0.71 | 0.24 to 1.18 |
|  |  |  |  | Unclear | 4 | 0.38 | 0.05 to 1.71 |
|  | Therapy | 6.87 | <0.01 | CBT | 3 | 0.72 | 0.44 to 0.99 |
|  |  |  |  | DBT | 2 | 0.16 | -0.14 to 0.47 |
|  | Treatment setting | 6.90 | 0.03 | Individual | 1 | 0.69 | 0.30 to 1.08 |
|  |  |  |  | Combined | 2 | 0.74 | 0.34 to 1.14 |
|  |  |  |  | Group | 2 | 0.16 | -0.14 to 0.74 |
|  | Control group | 2.50 | 0.11 | Active control | 3 | 0.31 | -0.04 to 0.66 |
|  |  |  |  | TAU | 2 | 0.74 | 0.34 to 1.14 |
| Global functioning | Therapy | 5.36 | 0.02 | CBT | 2 | 0.98 | 0.63 to 1.34 |
|  |  |  |  | DBT | 1 | 0.23 | -0.30 to 0.76 |
|  | Control group | 5.36 | 0.02 | TAU | 2 | 0.98 | 0.63 to 1.34 |
|  |  |  |  | Waiting list | 1 | 0.23 | -0.30 to 0.76 |
