# Supplementary material for: Long-Term Efficacy of Psychosocial Treatments for Adults With Attention-Deficit/Hyperactivity Disorder: A Meta-Analytic Review
Source: Front Psychol. 2018 May 4;9:638. doi: 10.3389/fpsyg.2018.00638 (PMC5946687; doi:10.3389/fpsyg.2018.00638)
Supplement: Supplementary file 14 [file Table_12.DOCX]

Supplementary Material

Long-term Efficacy of Psychosocial Treatments for Adults with Attention-Deficit/Hyperactivity Disorder: A Meta-Analytic Review

**Carlos López-Pinar^*^, Sonia Martínez-Sanchís, Enrique Carbonell-Vayá, Javier Fenollar-Cortés, Julio Sánchez-Meca**

*** Correspondence:**

Carlos López-Pinar

[carlopi@alumni.uv.es](mailto:carlopi@alumni.uv.es)

| Supplementary Table 12.  Subgroup analyses for within-subject outcomes. | | | | | | | |
| --- | --- | --- | --- | --- | --- | --- | --- |
| Outcome | Moderator | χ^2^ test | *p* value | Subgroup | *k* Studies | SMD | 95% CI |
| Total ADHD symptoms | Risk of bias | 1.86 | 0.39 | Low | 1 | 1.52 | 0.99 to 2.05 |
|  |  |  |  | Unclear | 4 | 1.12 | 0.83 to 1.41 |
|  |  |  |  | High | 9 | 1.11 | 0.73 to 1.50 |
|  | Therapy | 7.42 | 0.06 | CBT | 7 | 1.25 | 0.79 to 1.71 |
|  |  |  |  | DBT | 4 | 0.85 | 0.56 to 1.14 |
|  |  |  |  | MBCT | 1 | 1.55 | 1.02 to 2.08 |
|  |  |  |  | BFB | 1 | 1.55 | 0.81 to 2.29 |
|  | Treatment setting | 15.83 | <0.01 | Individual | 3 | 1.53 | 1.23 to 1.83 |
|  |  |  |  | Combined | 5 | 1.49 | 1.18 to 1.80 |
|  |  |  |  | Group | 5 | 0.80 | 0.52 to 1.07 |
|  | Outcome source | 0.23 | 0.63 | Self-report | 14 | 1.09 | 0.85 to 1.32 |
|  |  |  |  | Blind assessors | 5 | 1.18 | 0.90 to 1.46 |
| Inattention symptoms | Risk of bias | 8.23 | 0.02 | Low | 1 | 1.16 | 0.69 to 1.63 |
|  |  |  |  | Unclear | 2 | 0.88 | .70 to 1.05 |
|  |  |  |  | High | 5 | 1.46 | 1.09 to 1.83 |
|  | Therapy | 3.88 | 0.27 | CBT | 2 | 1.06 | 0.70 to 1.42 |
|  |  |  |  | DBT | 3 | 1.04 | 0.66 to 1.43 |
|  |  |  |  | MBCT | 1 | 1.63 | 1.08 to 2.18 |
|  |  |  |  | BFB | 1 | 1.40 | 0.69 to 2.11 |
|  | Treatment setting | 5.83 | 0.05 | Individual | 2 | 1.54 | 1.11 to 1.98 |
|  |  |  |  | Combined | 3 | 1.30 | 0.72 to 1.88 |
|  |  |  |  | Group | 3 | 0.95 | 0.71 to 1.20 |
|  | Outcome source | 3.88 | 0.05 | Self-report | 8 | 1.20 | 0.96 to 1.44 |
|  |  |  |  | Blind assessors | 3 | 0.91 | 0.74 to 1.07 |
| Hyperactivity/ impulsivity symptoms | Risk of bias | 3.84 | 0.15 | Low | 1 | 1.06 | 0.57 to 1.55 |
|  |  |  |  | Unclear | 2 | 0.62 | 0.46 to 0.78 |
|  |  |  |  | High | 4 | 0.94 | 0.42 to 1.45 |
|  | Therapy | 17.10 | <0.01 | CBT | 2 | 1.15 | 0.75 to 1.54 |
|  |  |  |  | DBT | 2 | 0.62 | 0.46 to 0.78 |
|  |  |  |  | MBCT | 1 | 1.43 | 0.94 to 1.92 |
|  |  |  |  | BFB | 1 | 0.34 | -0.09 to 0.77 |
|  | Treatment setting | 5.75 | 0.06 | Individual | 2 | 0.88 | -0.19 to 1.95 |
|  |  |  |  | Combined | 2 | 1.15 | 0.75 to 1.54 |
|  |  |  |  | Group | 3 | 0.64 | 0.49 to 0.79 |
|  | Outcome source | 1.09 | 0.30 | Self-report | 7 | 0.83 | 0.59 to 1.08 |
|  |  |  |  | Blind assessors | 3 | 0.67 | 0.49 to 0.87 |
| CGI | Risk of bias | 0.99 | 0.32 | Low | 1 | 0.97 | 0.50 to 1.44 |
|  |  |  |  | Unclear | 4 | 1.26 | 0.93 to 1.59 |
|  | Therapy | 0.25 | 0.62 | CBT | 3 | 1.29 | 0.73 to 1.85 |
|  |  |  |  | DBT | 2 | 1.13 | 0.83 to 1.42 |
|  | Treatment setting | 6.25 | 0.04 | Individual | 1 | 1.76 | 1.27 to 2.25 |
|  |  |  |  | Combined | 2 | 0.99 | 0.57 to 1.40 |
|  |  |  |  | Group | 2 | 1.13 | 0.83 to 1.42 |
| Global functioning | Therapy | 2.16 | 0.14 | CBT | 3 | 0.76 | 0.25 to 1.27 |
|  |  |  |  | DBT | 2 | 0.33 | 0.08 to 0.59 |
|  | Treatment setting | 8.92 | <0.01 | Combined | 3 | 0.84 | 0.51 to 1.17 |
|  |  |  |  | Group | 2 | 0.26 | 0.06 to 0.45 |
