# Supplementary material for: Long-Term Efficacy of Psychosocial Treatments for Adults With Attention-Deficit/Hyperactivity Disorder: A Meta-Analytic Review
Source: Front Psychol. 2018 May 4;9:638. doi: 10.3389/fpsyg.2018.00638 (PMC5946687; doi:10.3389/fpsyg.2018.00638)
Supplement: Supplementary file 16 [file Table_14.DOCX]

Supplementary Material

Long-term Efficacy of Psychosocial Treatments for Adults with Attention-Deficit/Hyperactivity Disorder: A Meta-Analytic Review

**Carlos López-Pinar^*^, Sonia Martínez-Sanchís, Enrique Carbonell-Vayá, Javier Fenollar-Cortés, Julio Sánchez-Meca**

*** Correspondence:**

Carlos López-Pinar

[carlopi@alumni.uv.es](mailto:carlopi@alumni.uv.es)

| **Supplementary Table 14.**  Meta-regressions for within-subject outcomes. | | | | | | | | | |
| --- | --- | --- | --- | --- | --- | --- | --- | --- | --- |
|  | Follow-up length | | | |  | % of participants on medication | | | |
| Outcome | Coeffi-cient | Standard error | 95% CI | *p* value |  | Coeffi-cient | Standard error | 95% CI | *p* value |
| Total ADHD symptoms (self-rated) | -0.01 | 0.03 | -0.08 to 0.05 | 0.68 |  | 0.00 | 0.00 | 0.00 to  0.01 | 0.28 |
| Total ADHD symptoms (blind assessor-rated) | 0.01 | 0.02 | -0.02 to 0.05 | 0.45 |  | 0.01 | 0.00 | 0.00 to  0.01 | <0.01 |
| Inattention symptoms (self-rated) | -0.02 | 0.03 | -0.08 to 0.05 | 0.64 |  | 0.00 | 0.00 | 0.00 to  0.01 | 0.58 |
| Inattention symptoms (blind assessor-rated) | 0.02 | 0.01 | -0.01 to 0.04 | 0.22 |  | 0.00 | 0.00 | 0.00 to 0.00 | 0.08 |
| Hyperactivity/Impulsivity symptoms (Self-rated) | -0.03 | 0.03 | -0.09 to 0.03 | 0.39 |  | 0.00 | 0.00 | 0.00 to 0.01 | 0.23 |
| Hyperactivity/Impulsivity symptoms (blind assessor-rated) | 0.00 | 0.02 | -0.03 to 0.03 | 0.99 |  | 0.00 | 0.00 | 0.00 to 0.01 | 0.68 |
| CGI | 0.05 | 0.02 | 0.00 to 0.09 | 0.07 |  | 0.01 | 0.00 | 0.00 to 0.01 | 0.01 |
| Global functioning | 0.23 | 0.15 | -0.06 to 0.51 | 0.12 |  | 0.02 | 0.01 | 0.01 to 0.04 | 0.01 |
